# Nutrition education improves dietary diversity of children 6-23 months at community-level: Results from a cluster randomized controlled trial in Malawi

**DOI:** 10.1371/journal.pone.0175216

**Published:** 2017-04-20

**Authors:** Judith Kuchenbecker, Anika Reinbott, Beatrice Mtimuni, Michael B. Krawinkel, Irmgard Jordan

**Affiliations:** 1 Justus Liebig University Giessen, Institute of Nutritional Sciences, Giessen, Germany; 2 Lilongwe University of Agriculture and Natural Resources, Bunda Campus, Lilongwe, Malawi; TNO, NETHERLANDS

## Abstract

Background: Low dietary quality and quantity and inappropriate feeding practices can cause undernutrition. Poor nutritional status in early childhood is associated with growth faltering. The objective of the study was to assess the potential of community-based nutrition education to improve height-for-age z-scores in children 6–23 months of age.

Methods and Findings: We carried out a cluster-randomized-controlled trial to assess the effectiveness of nutrition education. A total of 24 Extension Planning Area Sections served as clusters. The selection criteria were: the position of the extension officer was staffed and the sections had been selected by the project for activities in its first project year. The sections were randomized into intervention and control restricted on mean height for age Z-score using baseline information. In the intervention area, food security activities and community-based nutrition education was implemented. The control area received food security activities only. At baseline (2011) and endline (2014), caregivers with a child below two years of age were enrolled. Data assessment included anthropometric measurements, interviews on socio-economic status, dietary intake and feeding practices. A difference-in-differences estimator was used to calculate intervention effects. A positive impact on child dietary diversity was observed (B (SE) = 0.39 (0.15), p = 0.01; 95%CI 0.09–0.68). There was a non-significant positive intervention effect on mean height-for-age z-scores (B (SE) = 0.17 (0.12), p = 0.15; 95%CI -0.06–0.41). Limitations: The 24h dietary recalls used to measure dietary diversity did not consider quantities of consumed foods. Unrecorded poor quality of consumed foods might have masked a potential benefit of increased child dietary diversity on growth.

Conclusions: Participatory community-based nutrition education for caregivers improved child dietary diversity even in a food insecure area. Nutrition education should be part of programs in food insecure settings aiming at ameliorating food insecurity among communities.

## Introduction

Chronic malnutrition, reflected in stunting, is still a major problem among young children in Malawi [1–3]. Inadequate nutrition during early childhood is among the main contributing factors for stunting [4]. Further risk factors for impaired growth development are inappropriate breastfeeding as well as complementary feeding practices in children under two years of age [5,6]. There is evidence that not only food calories but dietary diversity (DD) is significantly associated with a child’s growth and weight [7,8]. Sufficient DD, meaning using a variety of foods to cover the nutritional needs of the growing child, is often not achieved in vulnerable populations.

To meet basic nutritional needs, the World Health Organization (WHO) recommends a consumption of at least a minimum of four out of seven different food groups per day for children 6–23 months of age, measured as minimum dietary diversity (MDD) [9]. Data from the most recent Malawi MDG Endline Survey 2014 showed that only 27% of Malawian children 6–23 months of age achieved MDD. In the same age-group, minimum acceptable diet (MAD) was achieved by only 15% of breast fed and 5% of non-breast fed children [3,9]. Besides inadequate DD, poor hygiene, and unhealthy practices, as well as insufficient knowledge of how to optimize DD using available resources for complementary feeding, contribute to high levels of child malnutrition [10,11].

Improving complementary feeding through nutrition education (NE) has been identified as a high impact intervention that could reduce stunting and its related burden of disease [12]. The concept of NE aims at voluntary adaption of food choices and food- and nutrition-related behaviors conducive to health and well-being. According to Contento, NE targets the individual but addresses also institutional, community and policy levels [13]. NE enhances people’s motivation to learn eating well and improves their ability and opportunities to do so [13]. Empirical evidence supports the importance of NE to improve feeding practices and accordingly also DD and child growth. Children of caregivers participating in a NE intervention in Kenya showed significant improvements of DD [14]. Also, NE significantly improved child dietary diversity (CDD), as well as mean intake of energy and selected nutrients in Ethiopia [15]. Combining NE with microcredit loans and entrepreneurship education significantly improved the height-for-age of children in Ghana [16]. All these studies assessed the impact of NE in actual participants compared to controls not receiving NE.

**Fig 1:**
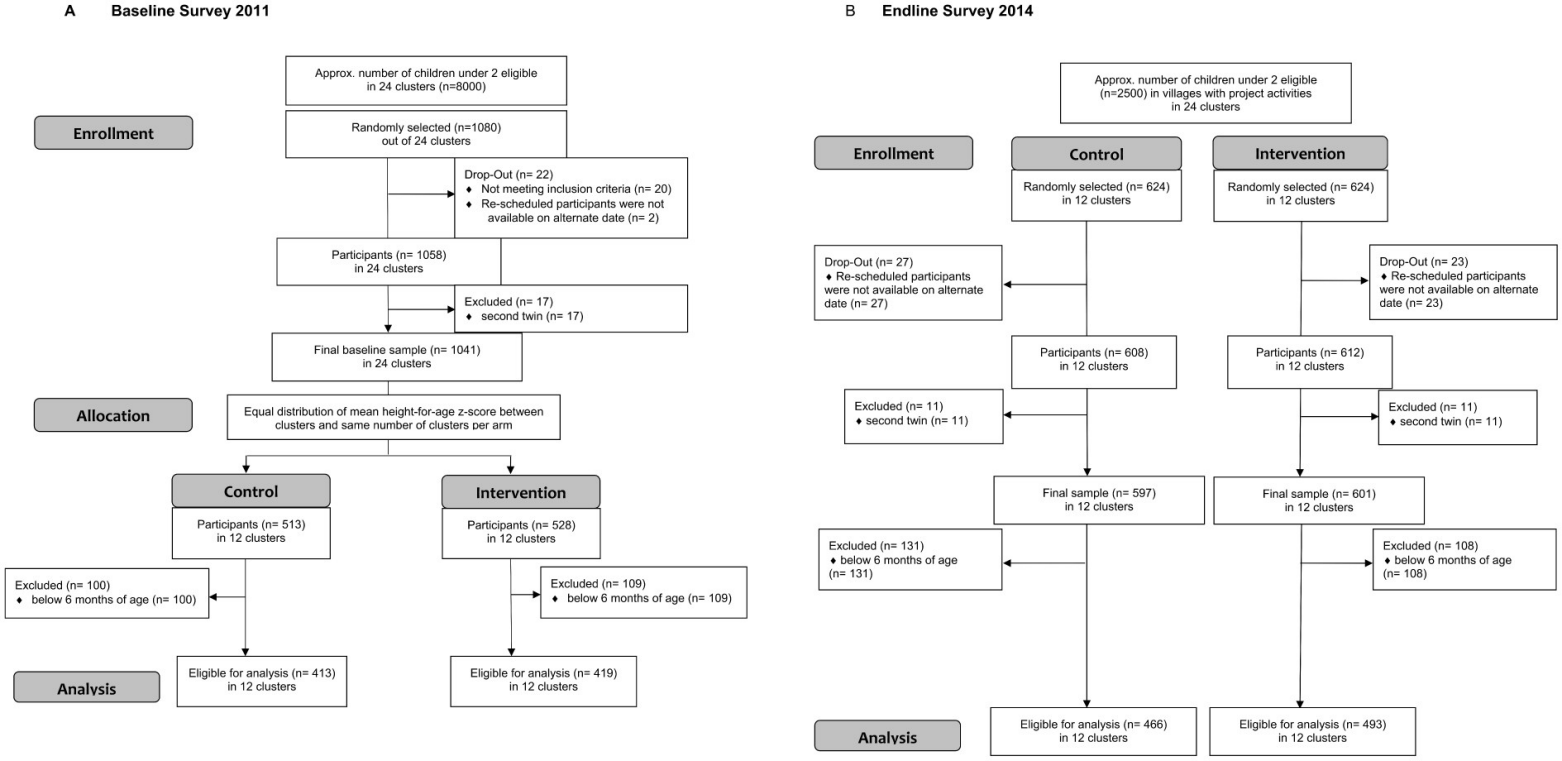
CONSORT flow diagram for (A) Baseline survey 2011 and (B) Endline survey 2014.

The project ‘Improving the dietary intakes and nutritional status of infants and young children through improved food security and complementary feeding counseling (IMCF)’ was designed to address the growing demand for evidence-based effective interventions to improve the nutritional status of children under two years of age. It comprised a NE intervention embedded into a food security project, implemented by local partners of the Food and Agriculture Organization of the United Nations (FAO), and a research project implemented by an independent academic research institution which evaluated the impact of the NE on children’s diet and nutritional status. A cluster design was chosen due to the implementing project’s approach which used the administrative boundaries of the Ministry of Agriculture of Malawi. (Fig 1)

**Fig 2:**
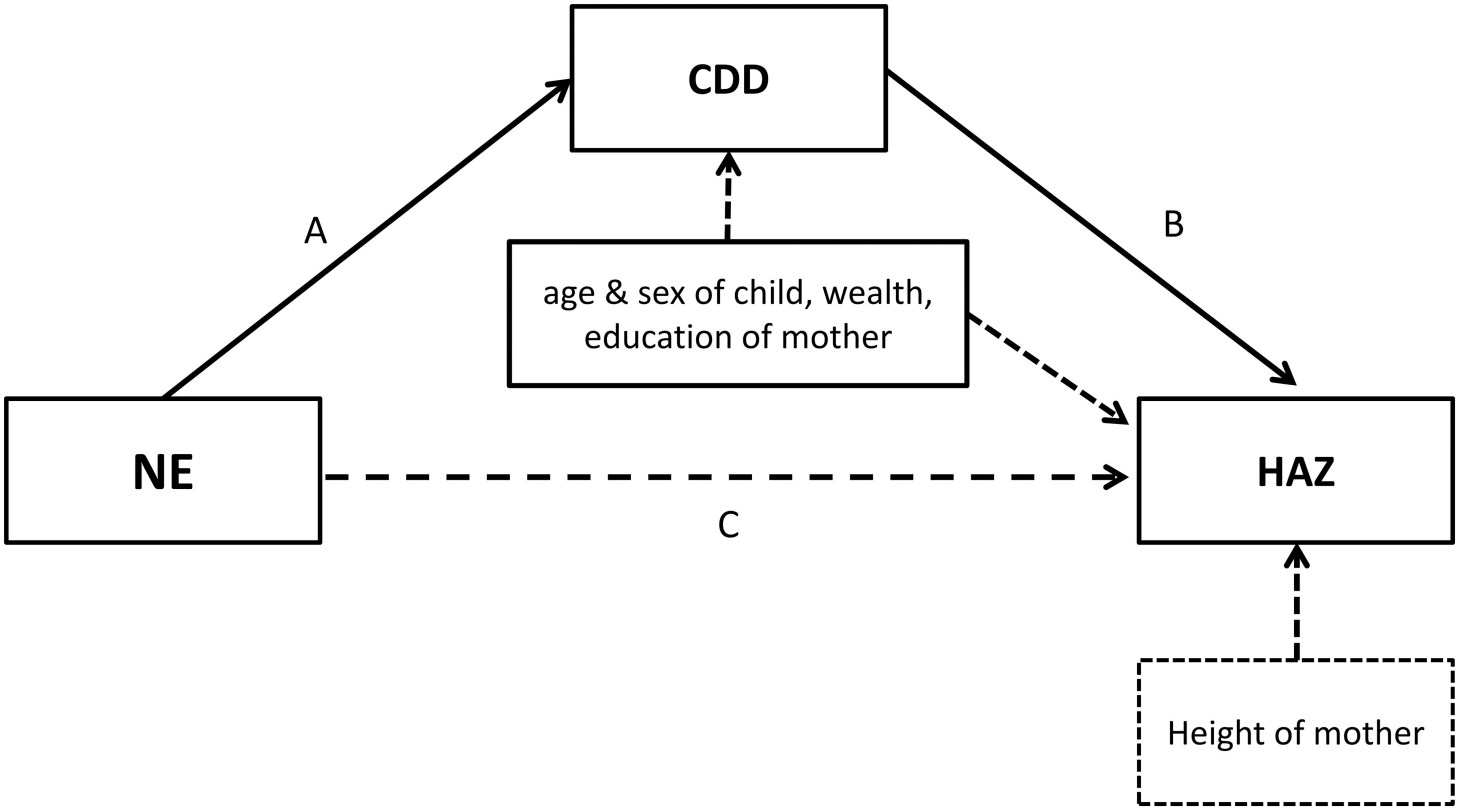
Indirect effects of nutrition education on HAZ. Pathway A: NE (the independent variable) impact on CDD (the mediator) controlled for age and sex of the child, wealth status of the household and educational status of the mother. Pathway B: HAZ (the dependent variable) influenced by CDD (the mediator) controlled for age and sex of the child, wealth status of the household, educational status and height of the mother. Pathway C: the model does not consider a direct effect of NE on HAZ.

The present study tested primarily the hypothesis that community-based NE led to improved height-for-age z-score (HAZ) and secondarily increased DD in children 6–23 months of age at cluster level in Kasungu and Mzimba districts in Malawi. The NE relied on trained village volunteers implementing NE sessions to a group of primary caregivers of children 5–18 months of age after social mobilization process. Whenever appropriate, fathers and grandmothers where invited to participate. The assumption was made that experiences made in the NE would be shared with community members who did not participate in the NE sessions. Further, it was assumed that this would lead to behavior change also among non-participants resulting in an overall improvement in infant and young child feeding (IYCF) practices and nutritional status of infants and young children in the targeted community. The NE was expected to increase child dietary diversity (CDD), and, higher CDD was expected to improve HAZ. Other pathways in which way NE could influence HAZ were not further evaluated in this analysis (Fig 2). In contrast to the studies described above, this study focused on the community effect.

## Methods

### Program background

Since April 2011, the Flemish International Cooperation Agency (FICA) funded the FAO food security project “Improving Food Security and Nutrition Policies and Programme Outreach” (IFSN) in a total of six extension-planning areas in Kasungu and Mzimba districts in the Central and Northern Regions of Malawi. Within these extension-planning areas, the IFSN project targeted a total of 24 sections in its first year which became the target area of the IMCF research project. Only sections where the position of an agriculture extension officer was staffed were included. The agriculture extension officers served as supervisor and trainers for the IFSN project. Thus, the sections served as clusters in the IMCF research design. To improve the overall food availability and diversity, IFSN implemented food security activities through Farmer Field Schools, Junior Farmer Field and Life Schools, and farmer field days from September 2011 –December 2014. Participants received inputs including seeds, fertilizer, fruit tree seedlings and livestock during this period.

Additionally, IFSN developed a participatory NE program “*Kadyetsedwe Koyenela Ka Ana”* considering feasibility and acceptability of improved complementary feeding practices and foods for children 6–23 months of age. The NE was field-tested and applied culturally acceptable and feasible IYCF practices. The program initiated NE groups where households were encouraged to include more local and seasonal food with emphasis on vegetables, fruits, pulses, and animal source foods (eggs, goat milk, small fish, etc.) to the diet of infants and young children. It was aligned to the Malawian Scaling Up Nutrition alliance which aims to reduce stunting among children under two years of age. The NE intervention was designed as a series of ten facilitated sessions (Table 1).

**Table 1:** Content of nutrition education (FAO 2014).

Session	Topic	Materials for nutrition education
Session 1:	Continuation of breastfeeding	Counseling cards
	Hand washing	Soap, ash, water in a jar or basin, model of tippy tap
	Food safety	Food storage containers and cups with covers
Session 2:	Complementary feeding ages (6, 7–8, 9–11, 12–23 months)	Counseling cards
	Porridge consistency	Recipe book
	Participatory cooking session one	Cooking utensils, firewood
		Ingredients for recipes
Session 3:	Malawi six food groups	Counseling cards
	Seasonal food availability calendar	Empty seasonal food availability calendar and set of food cards
Session 4:	Family meals and how they affect child nutrition	Counseling cards
	Participatory cooking session two	Recipe book
		Cooking utensils, firewood
		Ingredients for recipes
Session 5:	Vegetables, fruits and other healthy snacks	Counseling cards
		Recipe book
		Vegetables and fruits for food processing
Session 6:	Legumes and nuts	Counseling cards
	Participatory cooking session three	Recipe book
		Already processed soy and its products using different methods
		Unprocessed soy which mothers will learn to process
		Sprouted beans
		Un-sprouted beans which mothers will learn how to sprout
		Cooking utensils, firewood
Session 7:	Animal-source foods	Counseling cards
	Participatory cooking session four	Recipe book
		Cooking utensils, firewood
		Ingredients for recipes
Session 8:	Feeding the sick child, prevention, danger signs of illness	Counseling cards
		Invite HSA to come to the session
		Mothers should bring the health card of their child
Session 9:	Overall review of all the eight sessions and preparation for the graduation session	Counseling cards
Session 10:	Graduation	Food group examples
	Cooking session five	Examples of all recipes—as display for the community

Pairs of trained volunteers facilitated the NE sessions in their home villages among groups of 15 caregivers with children between 5–18 months of age. The materials were adapted from the UNICEF community based IYCF counseling cards template for Africa [17] and modified based on results obtained from two rounds of trials of improved practices [11]. The counseling cards are colored illustrations that depict key IYCF concepts and practices. The complementing facilitator’s book provides technical information about IYCF practices, and essential counseling instructions. The sessions covered topics on selection of age appropriate food, nutrients, diet, feeding children, food preparation (participatory cooking sessions), water, sanitation, and hygiene. The ten sessions were held weekly or bi-weekly for approximately 2–3 hours over a period of approximately five months. A new group of caregivers was formed after a break of about 3 months and the ten sessions were held by the same volunteers who received a one-day refresher training. Both rounds of NE were implemented in the second year of project implementation. Participation in the NE was voluntary. Food and firewood for cooking sessions was contributed by participants. At the end, mothers received a certificate during a graduation ceremony. Apart from this certificate, no incentives were given neither to the women nor children. The first round of the NE started in December 2012 followed by a second round starting in August 2013. Overall, the two rounds of NE included around 5430 women within the research villages.

### Design

The present study reports the analysis of a subsample of children 6–23 months of age with data from two repeated cross-sectional surveys in the project area: a baseline survey conducted in August/September 2011 and an endline survey conducted three years later in August/September 2014.

At baseline, the sample size was calculated based on stunting prevalence in children 0–23 months of age. This was done to study stunting prevalence and to measure mean HAZ in the study area. The mean HAZ values were needed for the randomization of the clusters in the upcoming trial. Prior to baseline, it was estimated that 8,000 children under two years lived in the research area. Considering 47% of children being stunted [2], a desired precision of ± 5% and a design effect of 3, the sample size calculation resulted in 1,096 children. A two-stage probability sampling strategy was applied. The EPA sections (clusters) were the primary sampling unit and for logistical reasons three villages per section were sampled. At the first sampling stage, villages were sampled proportional to population size using the software ENA for Smart^®^ [18]. At the second sampling stage, 15 households with children under two years of age were randomly selected from each village using the software R^®^ [19]. After the baseline assessment, the 24 EPA sections of the project area were allocated 1:1 to either the control or the intervention area. Equal distribution of HAZ, as well as numbers of control and intervention sections per district was considered as proposed by the research team. In each district, six sections were selected as intervention area, and six different sections served as controls. The control sections were “late-implementers”, meaning that the NE was scheduled to start after finalizing research activities in September 2014.

**Fig 3:**
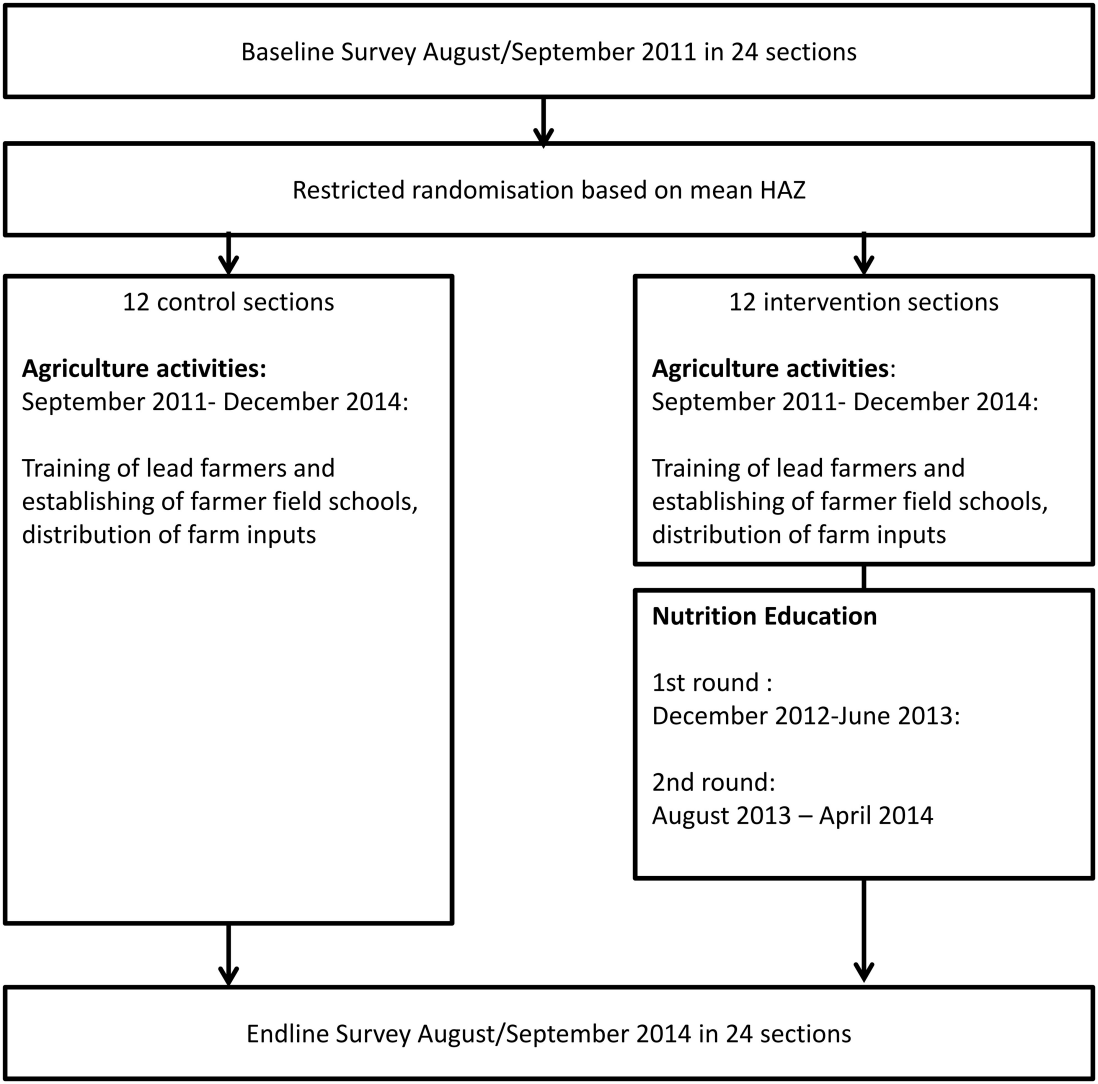
Flow-diagram of trial design. After a cross-sectional baseline survey in August/September 2011, the project area was divided in control and intervention area based on mean HAZ. From September 2011 –December 2014 the control area received agricultural activities only, while the intervention area received agricultural activities as well as two rounds of NE in the same time period. A cross-sectional endline survey was conducted in August/September 2014.

Three years after the baseline, an endline survey was conducted. By this point, the following program activities had been implemented through IFSN project staff, extension staff from the Ministry of Agriculture and Ministry of Health, as well as trained volunteers: Farmer Field School facilitator training and establishment of Farmer Field Schools, distribution of inputs (seed, fertilizer, farm assets, livestock) to selected IFSN-beneficiary households throughout the study area (intervention and control sections) as well as two rounds of the NE program in intervention sections only (Fig 3).

The sample size for the endline survey was estimated to examine the impact on HAZ in children 0–23 months of age. Sample size was calculated using the formula proposed by Hayes and Moulton for cluster randomized trials [20]. The calculation considered a power of 80% and a confidence level of 95%, μ_0_ − 1.69 (SD1.12) (mean HAZ at baseline), μ_1_ − 1.42 (SD1.12) (expected HAZ at endline based on an intervention effect of 16% improvement of HAZ). A design effect (DEFF) was included to adjust for intra-class correlation (ICC). DEFF was defined as: *DEFF* = 1 + *ICC*(*m* − 1). The ICC estimate—based on the baseline findings—was 0.025. The sample size per cluster (m) was set by 48 children under two years. Thus, the DEFF equaled 2.16. Multiplying the DEFF-value with the above calculation resulted in a required sample size of 582 children below two years of age for each arm. To account for non-responders, 10% were added resulting in a total sample size of 1,276 children under two years of age.

A two-stage probability sampling strategy was applied with sections being the primary sampling unit. In the intervention sections, only villages where NE was implemented since 2012 (= 181 villages) were included. In the control sections, only villages that had been targeted by agricultural activities of the IFSN project (= 203 villages) were included. Based on information from extension staff at cluster level, approximately 2500 children in the age group 0–23 months were living in the research area at endline. At the first sampling stage, four villages with probability sampling proportional to population size were selected per section using IBM^®^ SPSS^®^ version 20.0.0.2 [21]. At the second sampling stage, 13 households with children under two years of age were randomly selected in each village.

### Study setting and participants

Participation of the household in NE or other IFSN activities was not required for participation in the endline survey. Eligible participants were all permanent residents of the sampled villages with at least one child in the age range 0–23 months at the time of the survey. Primary caregivers with children were interviewed by trained local personnel in their native language, Chichewa or Chitumbuka.

### Ethics

The study was granted ethical approval by the Institutional Review Board of the faculty of medicine, Justus Liebig University of Giessen, Germany, and by the National Health Sciences Research Committee in Lilongwe, Malawi. Randomly selected participants were not coerced to engage in any study activities and an informed written consent on behalf of the children enrolled was sought from caregivers before any data were collected. For illiterate respondents, a thumb print was taken as signature. The participants were assured that they and their children would face no disadvantage in case they withdraw their approval to be enrolled for the study at any time. Confidentiality of the data and the privacy of participants were respected at all times. The study has been registered at the German Clinical Trials Register in Freiburg, Germany under the trial name: "Effectiveness of a nutrition education intervention to improve complementary feeding practices in Malawi: a restricted randomized trial " and the registration number: DRKS00003234.

### Data collection

A pre-tested, structured questionnaire in the respective language was used for data collection. Socio-economic variables, food security levels, children’s food intake as well as breastfeeding patterns, caregiver’s time budget, access to health care facilities, access to water and sanitation, and caregiver’s knowledge about food and feeding practices were assessed. Data collection on food and breast milk intake covered the previous 24hrs and was conducted as open recalls. The questions were designed according to Demographic and Health Surveys (DHS) [22] and WHO recommendations on IYCF [9]. Household wealth status was estimated following principal component analysis, the method used in DHS [23] including the following variables: Number of rooms used for sleeping per person, improved material of the roof, improved sanitation facility, size of land used for cultivation, and ownership of certain possessions (radio, cell phone, watch, mortar, machete, wheelbarrow, sprayer, ox-drawn implements, bicycle, ox-cart, chair, table, sofa). Food security was assessed by applying the Household Hunger Scale (HHS) [24] at baseline. Only households which answered the question if they were worried to not have enough food within the last four weeks were asked the complete set of HHS questions. Households which were not worried about their food situation were excluded from the HHS. At endline, food security was assessed with the Household Food Insecurity Access Scale (HFIAS) [25]. The present analyses used socio-economic data, data on food security, and children’s food intake. Weight and length of children was determined with the child wearing minimal clothing. Adults’ weights were taken while wearing light indoor clothing and no shoes. Heights and weights were assessed to the nearest 0.5 cm and 0.1 kg, respectively. All measures were taken twice and the mean was used for analysis. The maximum tolerated difference between the two measurements was: 0.7 cm for height/length, 0.1 kg for weight [26]. Weight was measured using standardized digital flat-scales (Seca 874, capacity: 200 kg, SECA GmbH & Co KG, Hamburg, Germany) with tara function. Recumbent length was taken from children using measuring boards (Seca 417, measurement range: 10–100 cm at baseline and Seca 416, measurement range: 33–100 cm at endline). The height of adults was measured with stadiometers (Seca 213, measuring range: 20–205 cm). Mid upper arm circumference (MUAC) was taken from pregnant women only.

### Statistics

Double data entry was performed for both data sets by different people using EpiData Entry version 3.1. [27] (baseline) and GNU PSPP version 0.8.2.1 [28] (endline). SPSS version 20.0.0.2 [21] was used for data cleaning. CDD was calculated based on a total of seven different food groups according to WHO specifications: (1) grains, roots and tubers, (2) legumes and nuts, (3) dairy products, (4) flesh foods, (5) eggs, (6) vitamin A rich fruit and vegetables, (7) other fruit and vegetables. To analyze the potential impact on CDD in more detail, comprising food groups of these seven food groups were analyzed resulting in a set of ten food groups: (1) grains, (2) roots and tubers, (3) groundnuts, (4) other legumes and nuts, (5) meat, (6) organ meat, (7) fish and seafood, (8) vitamin A rich roots and tubers, (9) vitamin A rich vegetables, (10) vitamin A rich fruit [9]. The WHO IYCF indicators MDD, MMF as well as MAD were calculated according to WHO guidelines [9]. HAZ, weight-for-age z-scores (WAZ), and weight-for-height z-scores (WHZ) were generated by using the most recent WHO growth standard data macros for SPSS version 3.2.2 [29]. Differences in socio-demographic characteristics and food groups consumed between intervention and control area were tested using t-test for continuous variables, χ^2^ test for nominal variables, and Mann-Whitney test for ordinal variables.

Stata^®^ 14.1 [30] was used to calculate a difference-in-differences (DiD) estimator to identify changes in outcomes associated with the impact of the program across the intervention sections. The DiD design is based on comparing two different groups. One of the groups was not affected by the intervention, i.e. not affected by the NE. The difference between the intervention group at baseline in 2011 (a) and at endline in 2014 (b) compared to the difference between the unexposed control group at baseline in 2011 (c) and at endline in 2014 (d) was calculated. The DiD is: (b-a)-(d-c), this way, the intervention effect can be estimated, controlling for baseline differences between the two groups and controlling for a general development over time. The underlying assumption of the DiD model is that the intervention group would have developed in a similar way to the control group, if they would not have receive any intervention [31]. In the present study, there is no reason to assume that the intervention group would have developed differently, given the study design. External factors which could have influenced the trend in the respective sectors differently were the same. Extension officers were employed by the government in all clusters throughout the project. All clusters were targeted by the program in the first year and there were no other relevant or unequally distributed activities in the groups by other agencies nor the government. Within the difference-in-differences (DiD) framework, HAZ, CDD and binary outcomes (MDD, MMF, MAD and food groups) were analyzed using linear probability models [32].

Cluster robust standard errors, allowing possible interdependencies between households of same villages, were used to get reliable and robust confidence intervals and *P*-values. The covariates height and education level of the mother, household wealth, as well as age and sex of the child were included into the model. To account for missing values, the estimation was done using full information maximum likelihood with the mlmv estimation method in Stata’s sem procedure. Only a few number of missing values were observed with a maximum of 9 missings in the indicators MMF and MAD at baseline (1.1% of baseline values) and a relatively large number in the covariate height of mother at baseline (30 of 832 = 3.6%) and endline (15 of 976 = 1.5%) since only the heights of biological mothers were taken. Since the missing values could be assumed to be missing at random and since no instrumental variables could be measured for the application of a multiple imputation (MI) model, FIML was preferred over MI for estimation [33].

The present analyses include children 6–23 months of age as a subsample based on the original sample size of children 0–23 months of age since the improvements of CDD through NE can only be measured in children ≥ 6 months of age. Further, analyses do not focus on the primary outcome that was formulated for the baseline survey (prevalence of stunting), but on the primary outcome: HAZ changes from baseline to endline. As a secondary outcome, the possible mediating impact of CDD on HAZ is analyzed. In contrast to the sample size calculation with HAZ as the primary outcome that included all children from 0–23 months we decided to analyze only the data of households and children from 6–23 months, because we regard the assumed mediating effect of CDD on HAZ as important to analyze. Thereby, the originally calculated sample size cannot be reached with this analysis resulting in an underpowered analysis. This is why non-significant results have to be evaluated carefully and the magnitude of the observed intervention effects needs to be interpreted substantially. Additional statistical tests were performed to describe and explore differences between intervention group and control group at the two time points (baseline and endline) and intervention effects on MDD, MAD, and single food groups. These statistical tests were not corrected for multiple testing and should only be interpreted in an exploratory way.

## Results

In total, 1,791 households with children 6–23 months of age participated in the surveys (832 at baseline, and 959 at endline, respectively). Main household characteristics of the study sample at baseline and endline stratified in intervention and control group are presented in Table 2. At baseline, 413 caregivers with children 6–23 months of age were enrolled in the control area and 419 in the intervention area. Three years later, the endline survey included 466 participating caregiver-child pairs in the control and 493 in the intervention area. There were no significant differences regarding household size and education of caregivers and household heads between the control and intervention area at baseline and endline. School education of caregivers was found six months longer at endline, school education of household heads remained stable. Wealth status of households was significantly higher in the intervention area at baseline. In both areas, wealth status decreased over time and was not significantly different at endline. According to the HHS applied to a subsample at baseline, more households in the intervention area experienced no or little hunger and less households suffered from severe hunger compared to the control area. At endline, food security was assessed with the HFIAS applied to the whole sample. Compared to the intervention area, more control households experienced severe food insecurity and fewer households were classified as food secure to mildly food insecure. Access to arable land was close to 100% in both surveys and the majority were farming households. Home garden ownership was significantly higher in the control area at both time points. However, home gardens declined over time by around 22% points in both areas. Less than 10% of the enrolled households were beneficiaries of inputs from the IFSN project at endline. Participation in farmer field schools was significantly higher in the intervention area. Access to improved drinking water increased over time by around 10%. Access to improved sanitation facilities was low at baseline and improved over time with higher improvements reached in the intervention area. Access to improved sanitation was significantly higher in the intervention area at endline. There were no differences regarding the sex of the household head (>90% male) or the marital status of the caregiver (>80% monogamously married) between control and intervention areas in both surveys.

**Table 2:** Household characteristics at baseline and endline.

	Baseline 2011 (n = 832)					Endline 2014 (n = 959)				
	Control		Intervention			Control		Intervention		
	**n**	**mean (±SD)**	**n**	**mean (±SD)**	***p***^+^	**n**	**mean (±SD)**	**n**	**mean (±SD)**	***p***^+^
Household size	412	5.8 (2.3)	419	5.7 (2.1)	0.49	466	5.3 (1.9)	493	5.3 (2.0)	0.78
Wealth status	413	-0.2 (3.4)	418	0.5 (3.7)	**0.01**	466	-0.3 (3.8)	493	0.1 (3.7)	0.10
Years of education of caregiver	412	5.9 (3.0)	418	6.2 (2.9)	0.12	466	6.5 (2.9)	493	6.8 (2.7)	0.09
Years of education of household head	409	8.1 (2.5)	416	8.1 (2.4)	0.79	449	8.1 (2.7)	480	8.3 (2.7)	0.41
	**n**	**%**	**n**	**%**		**n**	**%**	**n**	**%**	
Household food security*					0.32					<**0.01**
no—little hunger/food secure -mildly insecure	48	46.6	39	51.3		210	45.3	275	55.9	
moderate hunger/moderately food insecure	47	45.6	36	47.4		99	21.3	99	20.1	
severe hunger/severe food insecure	8	7.8	1	1.3		155	33.4	118	24.0	
Households with access to arable land	409	99.0	414	98.8	0.75	459	98.5	483	98.0	0.54
Households with home garden	339	82.3	319	76.1	**0.03**	285	60.9	266	54.0	**0.03**
Main source of income is farming	321	77.7	305	72.8	0.10	355	76.2	335	68.0	<**0.01**
Beneficiaries of food security inputs		n.a.		n.a.		34	7.3	38	7.7	0.82
Participants of farmer field schools		n.a.		n.a.		55	11.8	138	28.0	<**0.01**
Access to improved drinking water	310	75.2	315	75.2	0.98	392	84.1	429	87.0	0.20
Access to improved sanitation	128	31.0	113	27.0	0.21	201	43.4	253	51.4	**0.01**
Male headed households	385	93.7	387	92.4	0.46	426	92.2	453	92.1	0.94
Marital status of caregiver					0.57					0.90
married monogamous	335	81.3	347	82.8		377	81.3	400	81.1	
married polygamous	60	14.6	56	13.4		66	14.2	67	13.6	
widowed	1	0.2	4	1.0		6	1.3	2	0.4	
divorced or seperated	12	2.9	7	1.7		10	2.2	15	3.0	
single	4	1.0	5	1.2		5	1.1	9	1.8	

Number of missing cases 1–37 per variable: 1 (household size, wealth status, participation in farmer field school, access to improved drinking water, households with home garden, main source of income is farming), 3 (beneficiaries of food security inputs, Household food insecurity experience scale, marital status of caregiver), 5 (access to improved sanitation), 7 (male headed households) 20 (household food insecurity access scale), and 37 (years of schooling of household head).

*Household Hunger Scale was applied at baseline, including only households worried about their food situation within the previous 4 weeks (n = 176). Household Food Insecurity Access Scale was applied at endline for the whole sample (n = 959).

^+^ *p*-values from t-test for continuous variables, *χ*^2^ test for nominal variables, and Mann-Whitney test for ordinal variables

Table 3 summarizes the main characteristics of enrolled caregivers and children. Anthropometric data were collected if the caregiver was the biological mother of the enrolled child. There were no significant differences between intervention and control area at baseline and endline regarding age of the caregiver, weight, height, as well as body mass index (BMI) and BMI categories of mothers. Mean age of enrolled children was highest in the control area at endline, but was not statistically different from the intervention area. Regarding the anthropometry and sex of the enrolled children, there were neither significant differences at baseline nor at endline. WAZ was lowest at baseline in the intervention area and improved for both areas at endline. Compared to the baseline survey, HAZ was found lower in the control area while it remained unchanged in the intervention area. WHZ improved in both areas between the two time points. Both surveys included slightly more boys than girls in the intervention as well as in the control area. At baseline, significantly more children were currently breastfed in the intervention area. At endline, significantly fewer children in the intervention area suffered from diarrhea within the last two weeks prior the survey.

**Table 3:** Characteristics of primary caregivers and children at baseline and endline.

	Baseline 2011 (n = 832)					Endline 2014 (n = 959)				
	Control		Intervention			Control		Intervention		
	**n**	**mean (±SD)**	**n**	**mean (±SD)**	***p***^+^	**n**	**mean (±SD)**	**n**	**mean (±SD)**	***p***^+^
Age of caregiver (years)	401	27.2 (6.7)	404	26.7 (6.0)	0.22	454	26.7 (6.4)	488	26.2 (6.3)	0.18
Anthropometry of biological mothers										
weight (kg) (non-pregnant)	382	53.7 (7.1)	395	53.5 (8.1)	0.78	445	53.3 (7.2)	475	53.4 (7.3)	0.77
height (cm)	397	155.9 (5.6)	405	156.0 (5.5)	0.76	454	154.8 (5.7)	490	154.5 (5.7)	0.37
BMI (kg/m^2^) of biological mothers	364	22.1 (2.5)	389	22.0 (2.8)	0.49	445	22.2 (2.5)	475	22.4 (2.7)	0.28
	**n**	**%**	**n**	**%**		**n**	**%**	**n**	**%**	
BMI categories of biological mothers					0.54					0.37
underweight	10	2.7	29	7.5		14	3.1	19	4.0	
normal weight	318	87.4	311	79.9		387	87.0	395	83.2	
overweight	30	8.2	43	11.2		40	9.0	55	11.6	
obese	6	1.6	6	1.5		4	0.9	6	1.3	
	**n**	**mean (±SD)**	**n**	**mean (±SD)**		**n**	**mean (±SD)**	**n**	**mean (±SD)**	
Age of children (days)	413	445 (164)	419	458 (157)	0.24	466	478 (162)	493	461 (161)	0.09
Anthropometry of children										
weight (kg)	411	8.9 (1.5)	417	9.0 (1.5)	0.62	466	9.3 (1.5)	493	9.2 (1.5)	0.75
length (cm)	412	72.9 (5.8)	415	73.1 (5.6)	0.58	466	73.7 (6.0)	493	73.3 (5.8)	0.32
WAZ	411	-0.86 (1.1)	417	-0.93 (1.1)	0.40	466	-0.76 (1.05)	493	-0.69 (1.07)	0.27
HAZ	412	-1.71 (1.2)	414	-1.81 (1.1)	0.16	465	-1.85 (1.10)	493	-1.79 (1.15)	0.42
WHZ	411	0.03 (1.0)	414	0.01 (1.0)	0.76	465	0.27 (0.96)	493	0.32 (1.00)	0.38
	**n**	**%**	**n**	**%**		**n**	**%**	**n**	**%**	
Sex of children										
male	216	52.3	218	52.0	0.94	245	52.6	265	53.8	0.72
female	197	47.7	201	48.0		221	47.4	228	46.2	
Currently breastfed children	375	90.8	396	94.5	**0.04**	436	93.6	472	95.7	0.13
Episode of diarrhea within last 2 weeks	183	44.3	184	43.9	0.91	255	54.7	232	47.2	**0.02**

WAZ: weight-for-age z-score, HAZ: height-for-age z-score, WHZ: weight-for-height z-score. Number of missing cases 1–118 per variable: 1 (episode of diarrhea), 4 (weight of child, WAZ), 5 (length of child), 7(HAZ), 8 (WHZ) 44 (age of caregiver), 45 (height of mother), 94 (weight of mother), 118 (BMI of mother).

^+^ *p*-values from t-test for continuous variables, *χ*^2^ test for nominal variables, and Mann-Whitney test for ordinal variables

### Intervention effects

The consumption frequencies of the seven food groups used for measuring adequacy of young children’s diet by WHO are presented in Table 4. Nearly all children aged 6–23 months received grains, roots or tubers at baseline as well as at endline (>95%). Vitamin A rich foods, mainly green leafy vegetables, other fruit and vegetables, as well as legumes were the second most consumed foods in both surveys. Consumption of all animal source foods (ASF) was lower compared to plant-based foods in both areas and over time with flesh foods being consumed most followed by dairy and eggs. At baseline, consumption of legumes, vitamin A rich fruit and vegetables as well as other fruit and vegetables was significantly higher in the intervention area. Consumption of legumes was about 10% less in the control area at baseline and endline. For all ASF, consumption frequency in the control area was lower than in the intervention area at baseline and endline. Consumption of all ASF was significantly higher in the intervention area at endline. MMF and MAD were significantly higher in the intervention area at both time points. CDD did not differ at baseline but significantly improved in the intervention area compared to the control area at endline.

**Table 4:** Prevalence of food consumption and IYCF indicators at baseline and endline.

	Baseline 2011					Endline 2014						
	C	I		C	I	C	I		C	I	Intervention effect	
	(n = 413)	(n = 419)		(n = 413)	(n = 419)	(n = 466)	(n = 493)		(n = 466)	(n = 493)		
Food groups	prevalences unadjusted (%)		*p*^+^	prevalences adjusted* (%)		prevalences unadjusted (%)		*p*^+^	prevalences adjusted* (%)		(%)	*p*
**Grains, roots & tubers**	96.4	98.3	0.08	96.6	98.2	95.2	97.6	0.06	95.3	97.5	0.66	0.69
Grains	96.4	98.3	0.08	96.7	98.2	94.7	97.4	**0.04**	94.8	97.3	0.92	0.58
Roots & tubers	24.9	25.1	0.97	26.1	24.7	19.4	18.7	0.79	18.9	18.6	1.13	0.78
**Legumes & nuts**	57.1	67.5	<**0.01**	59.4	66.7	65.4	77.9	**0.01**	64.8	77.3	4.86	0.35
Groundnuts	35.8	46.3	<**0.01**	37.4	45.7	38.9	62.4	<**0.01**	38.7	61.9	14.96	<**0.01**
Other legumes & nuts	41.2	49.4	**0.02**	43.1	48.6	50.9	61.4	<**0.01**	50.5	60.9	4.87	0.34
**Dairy products**	12.9	13.4	0.83	13.5	12.5	15.6	20.9	**0.03**	16.2	20.6	5.47	0.15
**Flesh foods**	31.2	35.3	0.21	32.3	34.5	27.4	38.2	<**0.01**	27.0	38.4	9.20	0.09
Meat	15.5	16.7	0.64	16.0	15.9	12.8	18.9	**0.01**	13.0	19.1	6.18	0.11
Organ meat	2.7	2.8	0.80	2.8	2.3	1.3	2.6	0.14	1.2	2.6	1.96	0.16
Fish & seafood	19.9	23.2	0.25	20.6	22.9	18.5	24.1	**0.04**	18.2	24.1	3.57	0.43
**Eggs**	12.1	13.4	0.59	12.8	12.9	6.1	16.3	<**0.01**	6.1	16.2	9.92	<**0.01**
**Vitamin A rich fruit & vegetables**	69.0	75.7	**0.03**	71.1	75.6	71.6	77.5	**0.04**	70.2	77.3	2.62	0.53
Vitamin A rich roots & tubers	11.9	7.6	**0.04**	12.1	7.3	12.2	12.9	0.74	12.1	13.2	5.82	0.11
Vitamin A rich vegetables	65.9	73.0	**0.03**	68.0	72.9	67.8	73.3	0.07	66.4	73.1	1.77	0.67
Vitamin A rich fruit	10.4	16.2	**0.01**	10.8	16.1	6.7	16.9	<**0.01**	6.5	16.9	5.14	0.25
**Other fruit & vegetables**	72.4	78.3	<**0.05**	74.6	78.0	73.5	82.5	<**0.01**	72.5	82.0	6.16	0.11
**IYCF indicators**												
**MDD**	56.3	62.8	0.06	59.9	61.9	56.3	71.7	<**0.01**	55.5	71.1	12.70	**0.01**
**MMF**	80.0	89.2	<**0.01**	80.4	88.6	81.0	90.6	<**0.01**	81.6	90.3	0.43	0.91
**MAD**	47.9	57.7	<**0.01**	55.2	56.7	47.7	66.5	<**0.01**	47.3	65.8	11.86	**0.02**

C = Control, I = Intervention

*Prevalences adjusted based on DiD-estimates including covariates: wealth status, education of caregiver, age and sex of child

^+^ *p*-values from *χ*^2^ test for nominal variables

The DiD model adjusted for sex of the child, child’s age (days), education of caregiver (years) and wealth status showed a significant positive intervention effect on CDD (B (SE) = 0.39 (0.15), *p* = 0.01; 95%CI 0.09–0.68). The DiD estimated a mean CDD of 3.6 and 3.8 food groups for control and intervention area at baseline (p = 0.10). At endline, mean CDD decreased to 3.5 food groups in the control area and increased to 4.1 in the intervention area. All main food groups showed positive intervention effects ranging from 1–10 percentage points. Further disaggregated into comprising food groups, intervention effects ranging from 1–15 percentage points were achieved. The intervention significantly influenced consumption of eggs (p = <0.01), as well as groundnuts (p = <0.01). In the intervention area, consumption of eggs increased by 10 percentage points, consumption of groundnuts increased by 15 percentage points. The improvements in mean CDD in the intervention area at endline are further reflected in higher percentages of children reaching the WHO indicator MDD (BL = 61.9%, EL = 71.1%). Accordingly, within the control area, lower percentages of MDD (BL = 59.9%, EL = 55.5%) were achieved. There was no significant difference between both areas at baseline (*p* = 0.45). MMF was already high in both areas at baseline (C = 80.4%, I = 88.6%) however, significantly more children reached MMF within the intervention area (p = <0.01). There was almost no improvement regarding MMF at endline (C = 81.6%, I = 90.3%). The combined WHO indicator MAD showed a significant intervention effect of 12 percentage points at endline (p = 0.02) (Table 4).

The DiD model adjusted for sex of the child, child’s age (days), education of caregiver (years), wealth, and height of mother (cm) showed a non-significant, but small positive intervention effect on mean HAZ (B (SE) = 0.17 (0.12), *p* = 0.15; 95%CI -0.06–0.41). There was no significant difference between intervention (-1.87) and control area (-1.76) regarding mean HAZ at baseline (B (SE) = -0.12 (0.08), *p* = 0.17; 95% CI -0.28–0.05). **Including CDD as mediator variable** into the DiD model, confirmed that there was a small positive, but not significant intervention effect on mean HAZ that can be attributed to the mediator CDD (B (SE) = 0.01 (0.01), *p* = 0.15; 95%CI -0.01–0.02). Also, the association between CDD and HAZ was not significant (*p* = 0.06) (B (SE) = 0.04 (0.02); 95% CI 0.01–0.09.

## Discussion

This study examined the potential impact of a community based NE on growth of children 6–23 months of age via increased DD. While there was a positive and significant impact of NE on CDD, length of children did not increase significantly. At baseline in 2011, mean CDD was below the WHO recommended threshold of four out of seven food groups in both areas. At endline, children in the intervention area had significantly improved DD (p = 0.01) compared to children in the control area. This result demonstrates that the NE facilitated by trained local volunteers in ten sessions was effective at community level. The study by Waswa et al 2015 reported greater intervention effects on CDD with a fewer number of NE sessions (four sessions). However, all sessions were facilitated by the same professional nutritionist and only direct participants of the NE were assessed. In the present study, each sampled village in the intervention area had its individual pair of local volunteers, naturally influencing the facilitation and quality of the NE sessions with their personalities and qualifications.

Significant improvements of CDD can mainly be attributed to eggs and groundnuts. However, when having a detailed look of the intervention effect on egg consumption it has been noticed that consumption actually decreased in the control area by more than 50% of the baseline value and egg consumption increased slightly in the intervention area. Furthermore, eggs are relatively expensive and might not be affordable for most households regularly. Keeping this in mind, the NE still enabled households in the intervention area to increase egg consumption in children 6–23 months of age (BL = 13%, EL = 16%). While eggs are a very nutritious food it still has to be considered that no quantities were assessed and that it is common practice of mixing eggs with other food in a family pot. Consequently, the child might have received only a small portion of egg [34]. Regarding groundnuts, consumption increased in the intervention area while consumption remained stable in the control area. The improvement in groundnut consumption can be attributed to the intervention since groundnuts were also highly recommended in the NE. Moreover, groundnuts are produced all over the research area, are lower in price compared with eggs, and are eaten on a regular basis by all family members [35].

The WHO indicator MDD significantly increased in the intervention area at endline. On average, children in the intervention group achieved MDD while the DD was lower at endline compared to baseline in the control area, suggesting that the NE actually prevented a decrease in DD in the intervention group. The lower figures cited from the MDG Endline Survey as compared to the numbers in the current study might be explained as following. The dietary information in the MDG Endline Survey was captured by directly asking the participants if a food group was consumed rather than performing an open 24h dietary recall like in our study. Since quantities of foods are irrelevant to achieve a food group, a 24h recall probably captures more information. However, over- and underreporting have to be considered when assessing diets. Furthermore, dietary intake information in the MDG was captured in the lean season from November—April, whereas the current study assessed data in the post-harvest season assuming that food availability at household level was still sufficient. No validation of the caregivers responses were conducted.

However, despite the highlighted positive association of DD and growth of children found in other studies [7,8,16] our analysis revealed no significant association of DD and HAZ. The estimated change in HAZ in the DiD analysis was > 0.10 points lower than expected at the endline sample size calculation including children 0–23 months: B (SE) = 0.09 (0.11), *p* = 0.36; 95%CI -0.11–0.30, compared to an expected change of 0.27, respectively). The intervention effect in the study population including only children 6–23 months was 0.17 and still below the expected change of 0.27. Thus, the non-significant results cannot be satisfactory explained by the smaller sample size. The authors assume that a larger change in CDD is needed to observe a larger and then significant change in mean HAZ at community level. This might have been achieved if the nutrition education would have put extra emphasis on breastfeeding promotion considering the findings of the baseline study [5].

In the presented analysis, 10% of the observed non-significant positive intervention effect on HAZ can be attributed to CDD. The remaining 90% of the intervention effect cannot be explained with this analysis. A proposed reason for these findings might be the high prevalence of food insecurity throughout the study area at endline as well as a decline of the numbers of households with home gardens. Project activities aiming to improve food security in the intervention as well as the control area have obviously not been directed towards the target group of the NE: only 7.2% of households in the control area and 8.2% in the intervention area were immediate beneficiaries of food security inputs. Participation in farmer field schools was 11.6% in the control and 28.1% in the intervention area, respectively. Even if successful NE interventions on child growth have been reported for food insecure setting, NE as a single strategy was most effective in food secure populations [12,36].

Results from another study suggest that CDD does not mediate the relation between household food insecurity, stunting, and underweight [37]. Since the NE targeted children in the complementary feeding age only, caregivers of younger children were not included. This may have excluded a critical age group for the early development of growth retardation. As revealed by the baseline survey, exclusive breastfeeding was associated with higher mean HAZ as well as WAZ in children under six months [5]. The NE informed mothers about the benefits and importance of breastfeeding and encouraged mothers to continuing breastfeeding up to the child’s age of two years. Continued breastfeeding during pregnancy to avoid abrupt weaning was also emphasized. However, intensive promotion of exclusive breastfeeding of children 0–6 months of age was not part of the NE.

Furthermore, unrecorded poor quality of consumed foods might have masked a potential benefit of increased CDD for growth. Considering the increased consumption of groundnuts in the intervention group, also a potential negative impact of aflatoxins cannot be excluded. Groundnuts as well as maize (the main staple in Malawi consumed by >95% of all surveyed children) are highly prone to aflatoxin contamination. Various samples of maize and groundnut products including maize based baby foods and peanut based therapeutic foods were found with aflatoxin contamination above the EU maximum tolerable level [38]. Especially in Kasungu district, local farmers prioritize planting of tobacco and thereby delay planting of groundnuts. This exposes the crop to higher temperatures as well as end of season drought resulting in higher levels of contamination [39]. There is growing evidence that exposure to mycotoxins can impair child growth [40–42].

### Limitations

CDD was calculated based on mothers’ reported dietary intake which was not validated by another method. The questionnaire was pre-tested and enumerator training was detailed. The questionnaire as well as the 24h dietary recall and the interviewing technique were adapted from WHO’s indicators for assessing infant and young child feeding practices Part 2 Measurement. Single 24h dietary recalls as used to assess MDD do not consider quantities of consumed foods. It is likely that the consumed amounts of different foods were not sufficient to impact child growth. Although the present study demonstrates that DD can be increased with locally available resources, the actual nutrient adequacy or density of complementary feeding recipes was not assessed. Furthermore, detailed breast milk intake data would be needed to accurately estimate the nutrient requirement via complementary foods [43].

### Conclusion

The current study analyzed effects of a NE intervention on CDD and HAZ among children 6–23 months of age at community level. After adjusting for covariates, the intervention showed a significant positive effect on CDD. Dietary diversity stabilized and/or increased in the intervention area whereas it decreased in the control area. The intervention effect was mainly based on a higher consumption of eggs and groundnuts in the intervention area. Regarding food safety, future interventions promoting the consumption of groundnuts should consider and to measure aflatoxin contaminations of locally produced and stored groundnuts and to include education sessions about this challenge.

The absence of a significant effect on HAZ might be due to the lower sample size or chosen time between baseline and endline survey. Overall, food security seemed to be low in the study area, and this may have influenced the results. Nevertheless, the NE intervention increased CDD even in a food insecure setting. NE should be part of programs in food insecure settings aiming at ameliorating food insecurity among communities.

### Key messages

- Food security interventions and participatory nutrition education improved children’s dietary diversity at community level.
- Nutrition education facilitated by trained local volunteers in ten sessions was effective to improve IYCF practices.
- Linkage between agriculture and nutrition needs to be strengthened to further increase dietary diversity at household level.
- Food security activities should consider preventive measures to improve food safety, e.g. reduce the risk for mycotoxin contamination.

## Supporting information

S1 File IMCF study protocol. (PDF)

S1 Table Malawi minimal dataset. (XLSX)

S2 Table CONSORT 2010 checklist of information for a cluster randomised trial. (DOCX)
