# Supplementary material for: Nutrition education improves dietary diversity of children 6-23 months at community-level: Results from a cluster randomized controlled trial in Malawi
Source: PLoS One. 2017 Apr 20;12(4):e0175216. doi: 10.1371/journal.pone.0175216 (PMC5398527; doi:10.1371/journal.pone.0175216)
Supplement: S1 File — (PDF) [file pone.0175216.s001.pdf]

Effectiveness of a nutrition education intervention to improve complementary feeding practices in Malawi: a restricted randomized trial

Research Proposal Summary

Research question to be addressed by this proposal

The research question addressed by this study proposal is whether promoting improved complementary feeding recipes and messages developed through the formative research technique "Trials of Improved Practices (TIPs)" will have an impact on children's nutritional status and what changes in complementary feeding practices in the two districts, Kasungu and Mzimba, in Malawi can be identified. The overall aim of the study is to evaluate the effectiveness of the wider dissemination of improved infant and young child feeding practices developed by TIPs to improve child nutritional status.

Rational for Research

Malnutrition still remains one of the biggest challenges in developing countries. Children aged 0-23 months are the most vulnerable group with a peak incidence of mortality and morbidity. The promotion of a nutrient-dense diet based on locally available foods is essential to improve the nutritional status of young children. In order to optimize infants and young children's diets, FAO supports the formative research technique "Trials of Improved Practices (TIPs)". TIPs helps to understand families' preferences, capabilities as well as obstacles in improving their complementary feeding practices. Taking local circumstances into account, TIPs helps to develop nutrition information that are socially, culturally und locally acceptable and usable. The information acquired through TIPs are disseminated on a larger scale to improve complementary feeding practices region-wide.

The population of Malawi is facing various health and nutritional problems: 8% of the population is classified as food insecure, while at the same time non-communicable nutrition related disorders are becoming common. Knowledge on food utilization and dietary diversity are generally poor. Moreover, 12% of the population aged 15-49 is living with HIV/AIDS, about 64% of children aged 6-59 months suffer from anemia, and the estimated national stunting prevalence is about 47.1%. It is evident that stunting in children increases after they are six months of age when most children receive complementary feeding. FAO will therefore conduct TIPs in two districts in Malawi followed by a wider dissemination of behaviour change messages in order to reduce stunting and improve the nutritional status of children under the age of two.

The primary objective of the study is to show that children below two years have improved Height for Age Z-scores (HAZ) after at least 18 months of complementary feeding intervention, compared to children in matched control areas.

Secondary objectives are to investigate whether children in the intervention area have improved nutritional status measured by vitamin A and iron status and improved health status measured by incidence of acute respiratory infection (ARI) and diarrhea compared to children in matched control areas.

Methods

The overall study design is a cluster-randomized trial. This design enables to study the intervention (wider dissemination of TIPs results), which is not directed toward selected

individuals. Furthermore, it provides the ability to observe certain events across individuals, such as one individual's changing behaviors may influence another individual to do so.

Prior to the TIPs, a baseline survey will be conducted to assess the nutrition situation of families with children below two years in the FAO project region. After 18 months of intervention which will be conducted by the FAO/FICA project a cross-sectional impact survey will be conducted to evaluate, whether improved complementary feeding practices and recipes developed in the TIPs had an impact on the nutritional status among children below two years.

The surveys will include the following methods for data collection: **anthropometric measurements** and assessment of motor milestones and edema; **interviews** about the socio-economic situation, food security, mothers' and children's food intake, care, time availability, access to health, water and sanitation, and access to FAO food security activities (questionnaires are provided in the annex); collection of **capillary blood** to assess the micronutrient status (retinol binding protein (RBP), transferring receptor (TfR), hemoglobin, the morbidity status (C-reactive protein (CRP), acyl glycoprotein (AGP)) as well as Malaria, and **focus group discussions**. Hemoglobin concentration and Malaria status will be assessed immediately at the field site. The further analysis of blood samples will be done in Germany. Samples will be stored on ice in a high efficient styrofoam box with more than 5 cm thick walls and tightly closing lid. A member of the research team will deliver the blood samples to Germany.

Intervention: FAO/FICA will conduct TIPs in the assigned intervention areas in August 2011. The actual intervention will be the wider dissemination of improved recipes and behaviour change messages. During the dissemination phase data collection will include monitoring data on the dissemination and role out of the behaviour change messages through FAO/FICA project.

Population: Within the project areas all households with children 0-23 months are eligible to participate in the surveys. During the total data assessment period, families with children with a WAZ or WHZ-score <-2SD or sick children will be send for nutrition counseling and/ or treatment according to the countries guidelines. The study will not involve any special populations. Inclusion and exclusion criteria for participating in the surveys are: being resident in the sampled area, having at least one child 0 – 23 month of age, being randomly selected, accepting that anthropometric measurements and blood samples will be taken. Families with a child who does not have a written record of the child's date of birth or the date is not known by anyone in the family or who's age cannot be estimated based on a seasonal calendar of local events around one month will be excluded from the study. Eligible households will be found via village lists from the general population in the research area.

The sample size calculation resulted in 568 children below two for each treatment arm, considering a power of 80%, confidence level of 95%, $\sigma 0= 1.5$ and $\sigma 1=1.2$ and estimated 15% increase of HAZ ($\mu 0=-1.96$, $\mu 1=-1.66$) and an extra 10% to account for drop-outs or non-responders. Therefore, a total of approximately 2300 households with children below two years will be involved (baseline and impact assessment).

Risks / Benefits to Subjects

There are **no risks involved** while participating in the study.

The study does provide an opportunity for the participants to gain information about their and their children's current nutritional and health status. Participants will receive a "Participants-Card" including their and their children's health data available directly in the field (anthropometric data, age and hemoglobin level). The card will provide information whether the anthropometric measurements or the measured hemoglobin level indicate poor nutritional

status. In case of abnormal results participants will be send for nutrition counseling/ or treatment according to the guidelines of Malawi for treatment of anemia and malnutrition.

Costs and Compensation

Subjects will not receive any compensation and will not be asked to assume any out-of-pocket costs for participating in the research.

Confidentiality Assurance

All Investigators and study site staff involved with this study must comply with the requirements of the respective data protection laws in Malawi and Germany with regard to the collection, storage, processing and disclosure of personal information. Access to collated participant data will be restricted to the survey management and stored in a locked cupboard.

Each subject will be assigned a unique identification code that will be used for data entry and analysis. Test tubes and specimens used by laboratory staff will be labeled by individual code numbers only. The collected blood samples will only be used for the specific purpose covered by the informed consent given. Hardcopies of the data will be stored at Bunda college (responsible person: Dr. Beatrice Mtimuni 0888-851-870). Hardcopies will be disposed after a period of 10 years. Published results will not contain any personal data that could allow identification of individual participants.

Conflict of Interest

There is no actual or potential conflict of interest in relation to this research study.

Collaborative Agreements

An application for ethical approval for the research project has been submitted (04.05.2011) to the Institutional Review Board of the Justus Liebig University, Giessen, Germany. The letter of approval is expected in about six weeks.

Intended Use of Results

Dissemination of findings will involve regular monitoring of the project activities and a report every six months. This will contain information on main activities, compliance with the work plan and will identify problems and constrains. A contact information database will be established to facilitate communication between the Project management, Site Management, and the Technical Advisory Committee (TAC). The project visibility will be enhanced by establishing a website. Finally lessons learnt from the project and research results will be shared through participation and presentation in relevant conferences and technical consultations, preparation of research articles to be submitted to scientific journals, documentation of case studies as well as preparation of guidelines and technical recommendations on improved complementary feeding using local resources.

Effectiveness of a nutrition education intervention to improve complementary feeding practices in Malawi: a restricted randomized trial

Research Protocol

Bunda College:

Ph.D. Beatrice Mtimuni

M.Sc. Gabriella Chapota

Justus Liebig University:

Prof. Michael B. Krawinkel, MD

Dipl. oec. troph. Irmgard Jordan

M.Sc. Judith Kuchenbecker

April 2011

JUSTUS-LIEBIG-UNIVERSITÄT GIESSEN

UNIVERSITY OF MALAWI
BUNDA COLLEGE OF AGRICULTURE

Table of Contents

ABBREVIATIONS 4
1 Abstract 5
2 Background and justification 6
2.1 Trials of improved practices 6
2.2 Background information Malawi 7
2.2.1 FAO/ FICA food security and nutrition project in Malawi 9
2.3 Rationale of the Study 10
3 Literature review 10
3.1 Malnutrition and the role of food security for improving nutrition 10
3.2 The importance of complementary feeding 11
4 Hypothesis 12
5 Objectives 13
6 Methodology 14
6.1 Study sites 14
6.2 Participants 14
6.3 Study design 15
6.3.1 Overview of the study design 15
6.3.2 Trial design 15
6.3.3 TIPs and Intervention 16
6.4 Sample size for baseline and impact survey 16
6.5 Randomization procedure 17
6.6 Statistical methods and analysis 17
6.7 Ethical considerations 18
6.7.1 Recruitment, risks and benefits 18
6.7.2 Informed Consent 18
6.8 Data collection 19
6.8.1 Interview 19
6.8.2 Anthropometric measurements 19
6.8.3 Date of birth 20
6.8.4 Blood samples 20
6.8.5 Motor milestones 21

6.8.6 Data collected on TIPs and Intervention 21
6.9 Quality assurance 21
6.9.1 Statement of Compliance 21
6.9.2 Translation of Questionnaire 22
6.9.3 Recruitment of field staff 22
6.9.4 Training and monitoring of data collectors 22
6.9.5 Assurance of communication 23
6.9.6 Pretest 23
6.9.7 Registration of study 23
6.10 Data protection 23
7 Dissemination of findings 24
8 Personnel 25
9 References 26
10 Annexes 31
10.1 LOGICAL FRAMEWORK OF RESEARCH PROJECT 32
10.2 IMCF Malawi work plan 35
10.3 Particpants card 36
10.4 Intra-class correlation and minimal detectable effect size 37
10.5 Estimated Budget (operational costs) 38

ABBREVIATIONS

AED	Academy of Educational Development
AGP	Acid glycoprotein
Aids	Acquired immune deficiency syndrome
ARI	Acute Respiratory Infection
CRP	C-reactive protein
EPAs	Extension Planning Areas
EU	European Union
FAO	Food and Agriculture Organization of the United Nations
Fe	Iron
FEWSnet	Famine Early Warning Systems Network
FICA	Flemish International Cooperation Agency
GIZ	German Society for International Cooperation
HAZ	Height for Age z-Score
HH	Households
HIV	Human Immunodeficiency Virus
IMCF	Improving the dietary intakes and nutritional status of infants and young children through improved food security and complementary feeding counseling
IRB	Institutional Review Board
MDG	Millennium Development Goals
MDHS	Malawian Demographic and Health Survey
MNMS	Malawi National Micronutrient Survey
MTCT	Mother-to-Child Transmission
NCHS	National Center for Health Statistics (US)
NGO	Non-governmental organizations
RBP	Retinol Binding Protein
SMART	Standardized Monitoring and Assessment of Relief and Transitions
SUN	Scaling up nutrition
TfR	Transferrin Receptor
TIPs	Trials of Improved Practices
UNICEF	United Nations International Children’s Emergency Fund
USAID	United States Agency for International Development
Vit A	Vitamin A
WAZ	Weight for Age z-Score
WHO	World Health Organisation

1 Abstract

Malnutrition still remains one of the biggest challenges in developing countries. Children aged 0-23 months are the most vulnerable group with a peak incidence of mortality and morbidity. The promotion of a nutrient-dense diet based on locally available foods is essential to improve the nutritional status of young children.

In order to optimize infants and young children's diets, FAO supports the formative research technique "Trials of Improved Practices (TIPs)". TIPs helps to understand families' preferences, capabilities as well as obstacles in improving their complementary feeding practices.

The Malawi Demographic Health Survey 2010 estimated a national stunting prevalence of 47.1%. Results show that stunting increases after six months of age when most children receive complementary feeding. FAO will therefore conduct TIPs in two districts in Malawi followed by a wider dissemination of behaviour change messages. The objective of the research is to evaluate the effectiveness of the behaviour change messages.

Prior to the TIPs, a baseline survey will assess the nutrition security situation of families with children below two years in the FAO project region. The behaviour change communication strategies identified in the TIPs process will be implemented in the prior selected intervention areas by the FAO project. A cross-sectional survey will be conducted by a research team after at least 18 months of intervention evaluating the impact of the intervention by a research team.

The primary outcome parameters to be measured will be mean Height for Age Z-Scores (HAZ). Secondary outcome parameters will be vitamin A deficiency, iron deficiency and behaviour change. Methods for data collection will include anthropometric measurements, questionnaires, collection of capillary blood, and focus group discussions. Statistical analysis will compare primarily the difference of the mean Height-for-age Z-score in intervention and control areas.

2 Background and justification

2.1 Trials of improved practices

In order to support countries in their efforts to address problems of food insecurity and malnutrition FAO has been promoting improved complementary feeding in several countries[1] in the past years by teaching families how to enrich young children's diets using locally available nutrient-dense foods. In Afghanistan and Zambia FAO has assisted the Ministries of Agriculture and Health to improve complementary feeding through formative research using Trials of Improved Practices (TIPs – see box below). This approach has been especially set up by FAO to identify improved recipes and ideal messages for programming to improve feeding practices of women or caretakers of infants and young children. While all complementary feeding recipes and recommendations developed during TIPs are targeted to children 6 month of age and higher, it is important to note that exclusive breastfeeding up to 6 month of age is recommended during the TIPs process (1). It is part of FAO's efforts to integrate nutrition into agricultural and rural development activities and to foster linkages between the health and agricultural sector. Recommended dietary practices (including improved local recipes) have now been published in separate manuals for Afghanistan (2) and Zambia (3). Participatory cooking demonstrations using nutritionally improved recipes in conjunction with dietary counseling at household level to optimize young children's dietary intake have been introduced in various community development projects in these countries. FAO programmes using the TIPs approach to improve infant and young child and family nutrition are ongoing in Cambodia and Laos and are currently developed in Malawi within the FAO/ FICA[2] project.

Box: What are Trials of Improved Practices (TIPs)?

TIPs are a formative research technique used in programmes that promote behaviour change. The methodology has been well tested and validated, particularly with regard to health and hygiene behaviour, and has been used in various countries to develop nutrition behaviour change communication strategies, including infant and young child feeding practices(4-6).

Using TIPs, programme planners gain an in depth understanding of families' preferences and capabilities, as well as the obstacles they face in improving their nutrition and their motivations in trying new behaviours and practices. TIPs therefore allow programme planners to pre-test, adapt and evaluate the actual practices and recommendations in line with local circumstances and needs for eventual dissemination and promotion on a larger scale.

The TIPs can be divided into the three steps:

1. Investigation of food security, family feeding and child feeding practices, development of preliminary list of improved feeding recommendations and training of TIPs facilitators,
2. TIPs implementation: participants explore how to improve their child's health and nutrition,
3. TIPs evaluation and development of detailed plan for disseminating acceptable and feasible feeding recommendations and recipes.

[1] Afghanistan, Cambodia, Lao People's Democratic Republic and Zambia
[2] FAO/Flanders decentralized cooperation programme (GDCP/MLW/001/FLA)

Preliminary evidence from FAO programmes that have employed TIPs to improve infant and young child feeding have demonstrated the following:

1) families' interest in using an increased variety of locally available nutrient-dense foods accessible in different seasons to improve the nutritional adequacy of complementary foods,

2) the relevance of introducing basic nutrition, child feeding and food selection and preparation skills,

3) the acceptability and practical feasibility of using improved complementary feeding recipes in the family setting.

The recipes were designed to meet the Recommended Daily Allowances of children 6 to 23 months[3] with affordable locally available foods using the WHO Guiding Principles for Complementary Feeding of the Breastfeed Child (1). However, due to the absence of rigorous impact evaluation studies, up to now there is only anecdotal evidence of the impact of these improved complementary foods and feeding recommendations on feeding practices, children's dietary diversity, nutritional intake and anthropometric status identified through TIPs within a FAO project.

2.2 Background information Malawi

According to the 2010 Malawian vulnerability assessment about 8% of the population is classified as food insecure (7). In January 2011, FEWsnet Malawi identified 14 food insecure districts in southern Malawi, three receiving humanitarian food assistance interventions (8). Non-communicable nutrition related disorders such as overweight, obesity, hypertension and diabetes are becoming common and silently contributing to the mortality rate in the country.

Food utilization and dietary diversification are generally poor. Inadequate knowledge of food choices and combinations from the Malawi Six Food Groups[4] , childcare and optimal feeding practices, prevent households from maximizing the nutritional benefits of available foods. Families often lack appropriate skills and access to technologies for food preparation, preservation and storage, resulting in decreased quantity and quality of available food (9). Results from the MDHS 2010 show that 72% of the Malawian children below 6 months of age are exclusively breastfed[5] . Almost 90% of the infants of 6-9 months are given complementary foods (10).

In 2004, the overall prevalence of stunting was 48% and has decreased to 41% in 2010 (NCHS Reference) according to the National Statistics (10,11). Using the 2006 WHO Child Growth Standards (12) for analysis of the MDHS data the national stunting prevalence was 52.5% in 2004 and dropped to 47.1% in 2010 (10, personel communication), Preliminary results of the MDHS 2010 suggest that stunting in

[3] Currently zinc recommendations for children 12-24 month cannot be met with locally available foods. Further clarification is needed why recommendations are up to 10 x higher for 12-24 moth old children compared to 6-11 month old children.

[4] The six food groups are staples, animal products, legumes, vegetables, fruits, fats and oils

[5] Breastfeeding status refers to a "24 hour" period = yesterday and last night.

Malawi is influenced by the educational level of the mother since stunting decreases from 53%[6] among children from uneducated mothers to 39%[7] among children from mothers with secondary school education. The results show also that the prevalence of stunting increases after 6 months of age when children start complementary feeding (Table 1)(10).

Table 1: Prevalence of stunting (%) among children < 5 years in Malawi, MDHS 2010 (10)

**DHS 2010**	**Prevalence of stunting (%) (Height for age Z-Score < -2SD) ***		**n**
	**HAZ <-3SD**	**HAZ <-2SD**	
Children < 5 years	19.6	47.1	4849
< 6month	5.7	17.3	352
6-8 months	12.2	25.2	271
9-11 months	10.7	27.6	246
12-17 months	20.9	45.9	483
18-23 months	29.2	61.3	576
24-35 months	25.5	56.0	985
36-47 months	17.7	51.6	986
48-59 months	18.4	47.6	951

* WHO Growth Standard 2006

According to the MDHS 2010 64% of the children ages 6-59 months suffer from anemia defined as haemoglobin levels below 11 g/dl.

The MDHS 2004 (11) indicates that 12% of the population age 15-49 in Malawi is living with HIV/AIDS which is one of the highest national prevalence rates in the world. Heterosexual contact is the principal mode of HIV transmission, while mother-to-child transmission (MTCT) accounts for about 25% of all new HIV infections (11). According to the USAID Health Profile Malawi in 2010, HIV prevalence rates have not changed since 2004 (13).

It is evident that a HIV infection has a direct effect in worsening children's nutritional status (14,15). However, according to the study results of Nalwoga et al. (15) the population-level impact of childhood HIV infection on their nutritional status is limited if the HIV prevalence is low. There are no prevalence

[6] WHO Growth Standard 2006
[7] WHO Growth Standard 2006

rates available for pre-school children in Malawi. UNICEF estimates that 120,000 Malawian children between 0 and 14 years old were HIV-infected in 2009 (16). Surveillance of AIDS cases in Malawi indicates that very few children who were infected through mother-to-child transmission survive up to 15 years of age (11). Thus, an unknown number of HIV-infected children do not appear in the commonly used HIV prevalence statistics.

2.2.1 FAO/ FICA food security and nutrition project in Malawi

In 2008, the Flemish International Cooperation Agency (FICA) funded the FAO food security project 'Improving Food Security and Nutrition Policies and Programme Outreach" (FAO/FICA)[8] in a total of six extension-planning areas (EPAs) in Kasungu and Mzimba district. In August 2008, at the beginning of the project activities, a nutrition baseline survey was conducted covering the project area. The results showed that 47% of the households in Kasungu and 2% in Mzimba had a low dietary diversity (3 or less food groups of 12 food groups)(17). In order to improve the overall food availability and food diversity the project focused on strengthening the agricultural extension system through the establishment of farmer field schools, distribution of seeds and livestock, introduction of improved agricultural production methods, and introduction of improved irrigation systems.

In April 2011 the FAO/FICA project entered its second phase and will be implemented to March 2015. The project will continue to offer policy programme advisory services. This will be done through direct grass-root interventions to the improvement of the situation on an economically and environmentally sustainable basis for approximate 15,000 food insecure households in the previously and newly targeted 12 EPAs in Mzimba and Kasungu Districts[9]. Thereby it will contribute to achievement of the Malawian Government's development goals in terms of improving the food security and nutrition situation. The project will pay special attention to the lack of knowledge in Infant and Young Child Feeding (IYCF) Practices through conducting Trials of Improved Practices (TIPs – see chapter 2.1) to generate behaviour change messages and culturally acceptable complementary feeding recipes. The already available material on complementary feeding recipes for Malawi will be used in the preparatory phase of the TIPs. The I-Life project for example developed a "Complementary Foods Recipe Book for Malawian Children" funded by USAID (18). However, for these recipes there is neither information available if they were tested on feasibility by the target group nor if they are accepted in rural areas of Malawi.

The TIPs implementation phase starts with the community mobilization for TIPs and the selection of TIPs households within the FAO/FICA project area. An initial home visit is conducted to assess the households' food availability and dietary diversity, followed by one counseling visit and 2 follow up visits. After the evaluation phase the improved complementary feeding recommendations considered feasible by caregivers who participate in TIPs will be disseminated widely in the project area using interpersonal and participatory group learning approaches. Front line workers from the Ministry of Agriculture and Food security, Ministry of Health, Ministry of Education and non-governmental organizations involved in infant and young child feeding extension will be involved in promoting the adoption of the tested behaviour change messages and improved recipes. In addition, lead farmers

---

[8] GDCP/MSW/001/FLA

[9] Kasungu: Santhe, Lisasadzi, Ku-Chipala, Chulu, Kaluluma, Mkanakhoti; Mzimba: Vibangalala, Emfeni, Luwerezi, Mbawa, Champira, Khosolo

responsible for nutrition promotion will be selected and trained to facilitate improved complementary feeding participatory learning sessions at nutrition and other relevant community based groups. Furthermore caregivers who participate in TIPs will become peer educators who will pass on the behaviour change messages and improved recipes to friends, relatives and neighbours. Efforts of frontline workers and lead farmers for nutrition promotion will be reinforced by campaigns and other media such as posters, booklets or leaflets, charts, calendars and community radio spots. Special attention will be given to creating awareness of underexploited local resources and food processing and preservation technologies to improve community and family nutrition.

The expected outcome of the FAO/ FICA nutrition component is that the capability of target households will be strengthened to the extent that the diets of children below two years meet their nutritional needs by the end of the project.

2.3 Rationale of the Study

FAO needs to document the impact of the TIPs approach, capitalise on lessons learnt and refine recommendations to inform the design of future interventions and prioritise resource allocation. However, FAO is not a research institution and has therefore asked the Justus Liebig University to evaluate their TIPs approach within ongoing FAO food security projects i.e. the FAO/FICA project in Malawi. The expected outcome is an assessment of the effectiveness of promoting locally-available and affordable complementary foods to improve infant and young children's nutritional status based on TIPs within FAO food security projects.

3 Literature review

3.1 Malnutrition and the role of food security for improving nutrition

The burden of undernutrition in many developing countries continues to be high and slows the potential for individual, social and economic development. High rates of wasting and stunting among children under five years of age are a reflection of the serious challenges many developing countries are facing: inadequate access to and availability of healthy and diverse foods, improper feeding and caring practices, as well as poor health and hygiene conditions (9,19-21).

The food and financial crises of 2008 and 2009 have brought governments' attention to the importance of addressing food and nutrition security as a fundamental component of socio-economic development and political stability. This trend is reflected by efforts to reform the Committee on World Food Security[10] , the creation of the High-Level Task Force on Food Security[11] as well as donors' renewed interest in food and nutrition security (EU Food Facility; Spanish MDG-Fund on Children, Food Security

[10] The Committee for World Food Security (CFS) is the United Nations' forum for reviewing and following up on policies concerning world food security. It also examines issues which affect the world food situation. It was established as a result of the food crisis of the 1970s, upon recommendation from the 1974 World Food Conference.

[11] At the end of April 2008 the United Nations' Chief Executives Board established a UN System High Level Task Force (HLTF) as a temporary measure to enhance the efforts of the UN system and International Financial Institutions in response to the Global Food Security Crisis.

and Nutrition; USAID's Feed the Future; World Bank Scaling Up Nutrition (SUN) Framework, and the 63rd World Health Assembly Resolution on Infant and Young Child Feeding). Growing attention is given to the role of agriculture and to the linkages between agriculture and health in improving nutrition.

The conceptual framework of nutrition (figure 1) shows the different impact factors on nutrition security as well as food and health security, which may be applied on individual, regional and national level.

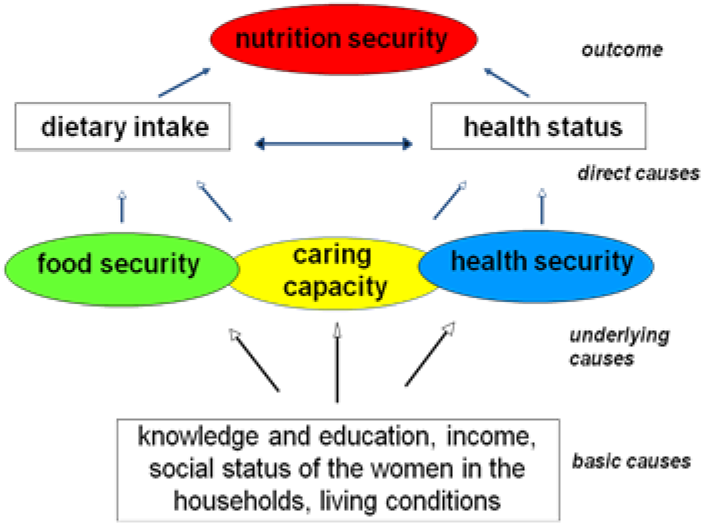

Figure 1: Adopted UNICEF framework of underlying causes of malnutrition and mortality (22,23)

The linkage between food production and nutritional health can only be achieved if the interactions among food diversity on the plate, people's nutritional knowledge and preferences, socio-economic factors, and crop diversity are considered (24).

There has been a demand for in depth research in these areas for many years (25) in order to inform policy makers and programmers to ensure funds made available for nutrition interventions are optimally utilized (26).

3.2 The importance of complementary feeding

Several reviews of nutrition interventions (27-29) have shown that increased attention needs to be given to complementary feeding interventions targeted to children aged 6-23 months, which is the period with the peak incidence of growth faltering, micronutrient deficiencies and infectious diseases in developing countries (30). The effects of poor nutrition resulting in stunting may also be associated with delayed motor and mental development (31,32). Therefore, effective interventions that are preventing and reducing stunting during this vulnerable period should be a high priority.

Several interventions, targeting this age group, put the emphasis mainly on micronutrient supplementation and food supplements, as well as therapeutic feeding and care (33-48). A meta-analysis looking at the impact of micronutrient interventions revealed a limited positive effect on height gain per year with less than 0.1 cm/year through either single or multiple nutrient supplementation compared to controls (42). However, studies focusing on complementary feeding support and educational strategies have shown to have a larger impact. Pooled analysis of three studies focusing on nutritional education in food-secure populations showed an increase in mean Height for Age Z-score (HAZ) of 0.25 (95% CI 0.01-0.49) compared to the control group. The effect was even higher in studies in food insecure populations receiving food supplements with or without education (HAZ change 0.41, 95% CI 0.05-0.76) (27). An evaluation study in Haiti comparing the impact of recuperative and preventive health services to comparable households in the Haiti Demographic Health survey on linear growth confirmed these results. In addition, the study showed that the preventive program had had a greater impact than the recuperative program compared to the respective control group (mean HAZ difference: 0.341; 95% CI 0.104, 0.577 and mean HAZ difference: 0.183; 95% CI: -0.022, 0.388 respectively) (49).

Comprehensive food-based approaches that promote a variety of nutrient-dense local foods to improve children's dietary intake and nutritional status are essential (9,50). Food-based approaches that focus on the use of locally available, affordable, and accepted nutrient-dense foods and recipes are designed to empower local populations to optimally use their resources and limit their dependency on external resources. Therefore, these approaches have a higher potential to improve child nutrition in the longer term compared to programs that mainly rely on donor funding and the distribution of micronutrients or food supplements.

4 Hypothesis

Assuming that behavior change messages on breastfeeding and complementary feeding practices as well as hygiene aspects as generated by the TIPs are widely accepted and out into practice by the mothers with children ages 0-23 months the data assessment will be based on the following hypotheses:

1. The Height for age Z-Score (HAZ) of children below 2 years will increase if TIPs recommendations and recipes are locally available and accepted.

   At conclusion of the intervention, there will be a difference of 15% of the HAZ between interventions and controls:

   H0: µ HAZ (intervention) = µ HAZ (control)

   H1: µ HAZ (intervention) > µ HAZ (control)

2. Secondly, prevalence of anemia, vitamin A deficiency as well as morbidity rates will be reduced.

   a) Anemia (A) among children between 6 and 23 months, defined as hemoglobin level of < 11g/dl, will decrease by 25 %.

   H0: P(A| intervention) = P(A| control),

   H1: P(A| intervention) < P(A| control)

b) Retinol binding protein (RBP) levels will decrease by 15% among children with a C-reactive protein (CRP) level of < 12mg/l.

H0: µ RBP (intervention) = µ RBP (control),

H1: µ RBP (intervention) < µ RBP (control)

c) Prevalence of children with presumed acute respiratory infections and fever (ARI) and diarrhea (D) in the last two weeks prior the survey will decrease by 25%.

H0: P(ARI| intervention) = P(ARI| control),

H1: P(ARI| intervention) < P(ARI| control)

H0: P(D| intervention) = P(D| control),

H1: P(D| intervention) < P(D| control)

d) There will be differences in knowledge of age-appropriate complementary feeding (KCF) of children between caretakers measured using a knowledge score in interventions and controls.

H0: µ KCF (intervention) = µ KCF (control),

H1: µ KCF (intervention) > µ KCF (control)

e) TIPs formative research generates nutritionally improved, culturally acceptable and affordable recipes which result in improved feeding practices and food intake which will result in a higher dietary diversity score for the children

There will be differences in dietary diversity (DD) between interventions and controls:

H0: µ DD (intervention) = µ DD (control),

H1: µ DD (intervention) > µ DD (control).

5 Objectives

The purpose of this study is to observe the impact of promoting improved complementary feeding recipes and messages developed through TIPs on children's nutritional status and identify changes in complementary feeding practices in the two districts, Kasungu and Mzimba, in Malawi. The overall aim of the study is to evaluate the effectiveness of the wider dissemination of improved infant and young child feeding practices developed by TIPs to improve child nutritional status.

The primary objective of the study is to show that children below two years have improved Height for Age Z-scores (HAZ) after at least 18 months of complementary feeding intervention, compared to children in matched control areas.

Secondary objectives are to investigate whether children in the intervention area have improved nutritional status measured by vitamin A and iron status and improved health status measured by incidence of acute respiratory infection (ARI) and diarrhea compared to children in matched control areas.

6 Methodology

6.1 Study sites

The intervention and control areas will be selected following the agricultural extension structure of the area covered by the FAO/FICA project. Therefore, 6 EPAs in Kasungu district (Chulu, Kaluluma, Mkanakhoti, Santhe, Lisasadzi, Ku-Chipala) and 6 EPAs in Mzimba district (Emfeni, Khosolo, Luwelazi, Champira, Vibangalola) will be included. These 12 EPAs can be divided into 135 extension sections served by x extension officers (x=number is currently assessed). The extension officers are responsible for a total of 10,398 villages with about 240,000 farm households. The study sites for the research will be the villages in the extension sections.

6.2 Participants

Knowing that recommendations on exclusive breastfeeding are included into the behaviour change strategies all households with children 0-23 months of age are eligible to participate in the surveys. During the total data assessment period, families with children with a WAZ or WHZ-score <-2SD or sick children will be send for nutrition counseling and/ or treatment according to the countries guidelines.

Inclusion and exclusion criteria for participating in the surveys are:

- being resident in the sampled area,
- having at least one child 0 – 23 month of age,
- being randomly selected,
- accepting that anthropometric measurements and blood samples will be taken.

Families with a child who does not have a written record of the child's date of birth or the date is not known by anyone in the family or who's age cannot be estimated based on a seasonal calendar of local events around one month will be excluded from the study (see chapter 6.8.3).

6.3 Study design

6.3.1 Overview of the study design

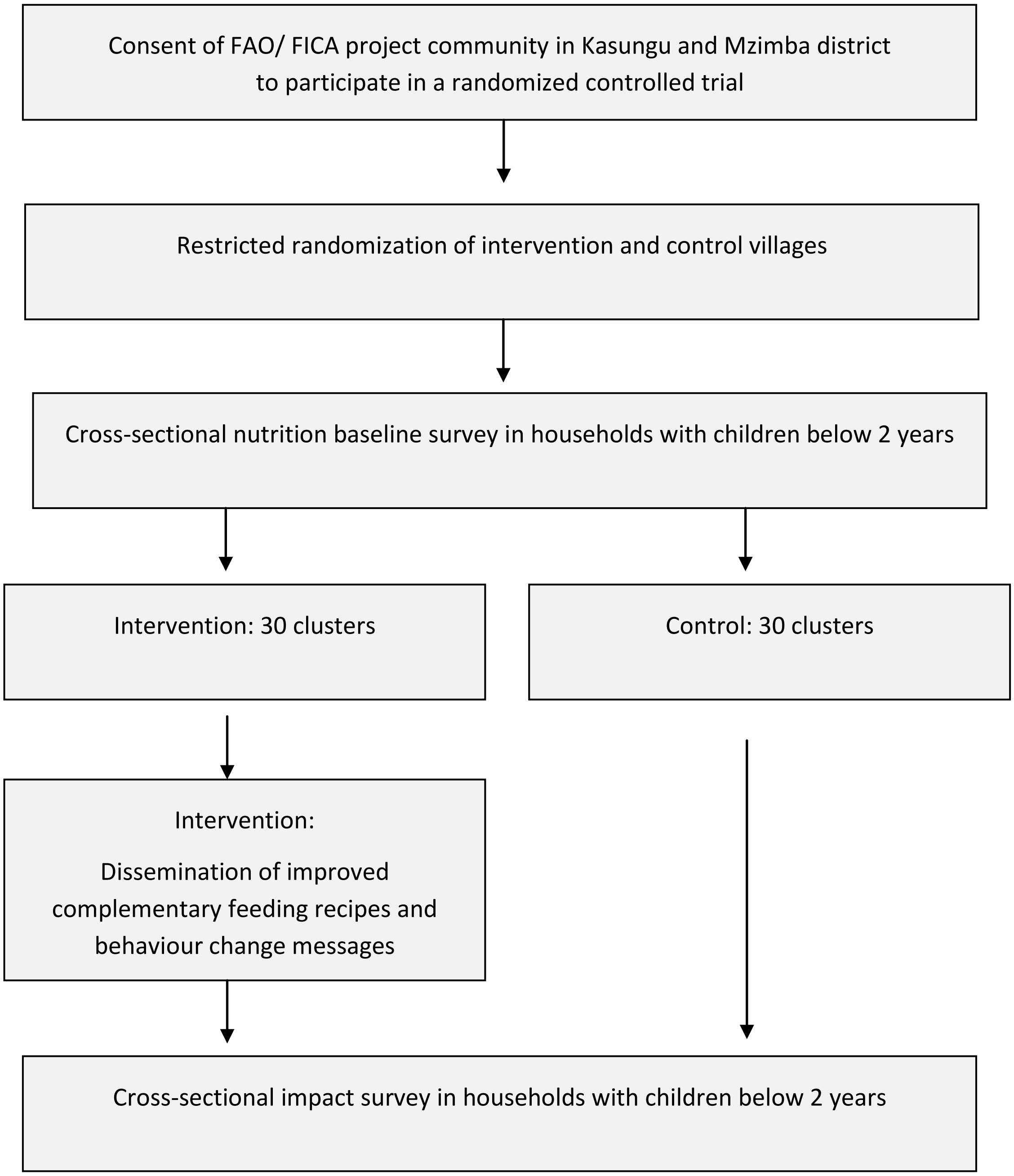

6.3.2 Trial design

Restricted randomization will be conducted to identify intervention and control areas. In July 2011, a baseline survey will be conducted to assess the outcome indicators prior to the intervention in the selected areas. The FAO/ FICA project will implement TIPs in selected households in the intervention area only followed by a wider dissemination of the behaviour change messages and improved

complementary feeding recipes. After at least 18 months of intervention an impact survey will be conducted that has been scheduled for August 2013.

After 18 months of intervention which will be conducted by the FAO/FICA project a cross-sectional impact survey will be conducted to evaluate, whether improved complementary feeding practices and recipes developed in the TIPs had an impact on the nutritional status among children below two years. The final survey is scheduled for August 2013.

6.3.3 TIPs and Intervention

FAO/FICA will start TIPs in the assigned intervention areas in August 2011[12] . The actual intervention will be the wider dissemination of the improved recipes and behaviour change messages, which will start as soon as the recipes and behvaiour change messages are available. During the dissemination phase data collection will include monitoring data on the dissemination and role out of the behaviour change messages through FAO/FICA project.

6.4 Sample size for baseline and impact survey

The appropriate sample size for cross-sectional surveys will be determined largely by three factors (51):

a) estimated prevalence of the variable of interest,

b) desired level of confidence and

c) acceptable level of error.

The cross-sectional surveys sample size will be based on the formula proposed by Haynes and Moulton for cluster randomized trials Haynes and Moulton (52):

$$n = (z_{\alpha/2} + z_{\beta})^2 \frac{(\sigma_0^2 + \sigma_1^2)}{(\mu_0 - \mu_1)^2}$$

The sample size calculation resulted in 335 children for each treatment arm, considering a power of 80%, confidence level of 95%, σ_0= 1.5 and σ_1=1.2 and estimated 15% increase of HAZ (μ_0=-1.96, μ_1=-1.66)[13].

Nevertheless, to adjust for intra-class correlation (ICC), a design-effect should be included. The design effect is defined as

$$DEFF = 1 + ICC(m-1)$$

Considering that m=19 children per cluster (village) will be measured, and an estimated ICC of 0.03 is applied (see annex 10.3), the DEFF= 1 + 0.03*18=1.54. Multiplying the DEFF-value with the above

[12] The research team from JLU/Bunda will observe the TIPs (see 6.8.1.).

[13] Values have been estimated from the available literature.

calculation this results in 516 children below two for each treatment arm. Adding an extra 10% to account for drop-outs or non-responders results in a sample size of 568 children below two years for each treatment arm.

6.5 Randomization procedure

The intervention and control villages are assigned following a restricted randomization. This implies that randomization will take place after adjusting for certain variables that might interfere with variables of interest to the study. Variables of adjustment are:

- extension officer
- access to health posts, nutrition rehabilitation unit, or nutrition counseling by a NGO
- total village population
- average land holding size
- ethnic distribution
- access to markets, and
- project activities by FAO/FICA.

The selected clusters (villages) will be the objective of randomization. Randomization will be done using the Software package “Experiment” and the operation “randomize”. The “Experiment”-package is a software extension to the statistical software R©. It serves to design and analyze different types of randomized trials, including cluster randomized trials, block, or matched pair designed trials (53).

The baseline and the impact survey will randomly select the study participants in the selected clusters (villages).

6.6 Statistical methods and analysis

Data assessment and data analysis will follow the adopted UNICEF framework of underlying causes of malnutrition and mortality (see chapter 3.1). Nutritional status will be interpreted as a result of household food security, i.e. access to food and food availability, adequate social and care environment, i.e. direct caring behaviours, women’s role, status and rights, social organizations and networks, and functioning public health system, i.e. health environment and access to health.

The cross sectional surveys will be used to gather information of the above mentioned parameters before and after the intervention. The data will be entered into the SPSS editor (IBM SPSS Statistics version 19) and checked for inconsistencies by two individuals independently. Flagged values will be

checked based on the filled questionnaires and if applicable the study participants will be approached to verify the result.

Statistical analysis will be performed using the statistical packages of IBM SPSS statistics (version 19). At first the variables will be tested for normal distribution, followed by a descriptive analysis of prevalence of malnutrition, anemia, vit-A deficiency, respiratory infections and fever (ARI), diarrhea, morbidity rates, dietary diversity, and knowledge of age appropriate feeding. The latter involves a development of a score to describe the knowledge about age appropriate feeding. Further, regression analysis will be applied looking for causalities of malnutrition in the studied area.

Focus groups discussions will be based on a general interview guide approach. The interview guide will include subjects of implementation, acceptance and obstacles of TIPs. They will be analyzed by applying the content analysis according to Mayring (54) and facilitated by the use of MAXQDA©-software (55).

6.7 Ethical considerations

Ethical approval will be obtained from the Institutional Review Board (IRB) of the University of Giessen and local National Health Science Research Committee in Malawi prior to commencement of the study.

6.7.1 Recruitment, risks and benefits

Lists of possibly eligible participants will be obtained from official population’s lists.

Risk to participants: There are no risks involved while participating in the study.

Benefits to Participants: This study has an indirect benefit to the participants. The study does provide an opportunity for the participants to gain information about their and their children’s current nutritional and health status. Participants will receive a “Participants-Card” including their and their children’s health data available directly in the field (anthropometric data, age and hemoglobin level). The card will provide information whether the anthropometric measurements or the measured hemoglobin level indicate poor nutritional status. In case of abnormal results participants will be sent for nutrition counseling/ or treatment according to the guidelines of Malawi for treatment of anemia and malnutrition.

6.7.2 Informed Consent

Prior to the surveys, general consent of the FAO/ FICA project communities to participate in this trial will be obtained. Written consent will be obtained by the individual household to be interviewed at the day of data assessment.

The Investigators will be responsible for ensuring informed consent is obtained before any protocol specific procedures are carried out. The decision of a participant to participate in the research study is voluntary and will be based on a clear understanding of what is involved. Participants will receive adequate oral and written information about the nature and purpose of the study, participation/termination conditions, and risks and benefits – appropriate Participant Information and Informed Consent Forms are provided in the Annex. The oral explanation to the participants will be

performed by designated people (enumerators), and must cover all the elements specified in the Participant Information Sheet and Informed Consent Form.

The participants will be given every opportunity to clarify any points they do not understand and, if necessary, ask for more information. The participant will be given sufficient time to consider the information provided.

The enumerator and the participant have to sign and date the Informed Consent Form to confirm that consent has been obtained. The participant will be provided with a copy of this document.

6.8 Data collection

The data collection in the cross sectional surveys will be based on an adaptation of the SMART methodology (51). The SMART methodology includes a questionnaire designed to assess mortality, nutritional status and food security in crisis situations. The final questionnaire will consist of standardized modules extracted from the questions designed by the SMART initiative, FAO, WHO, GIZ, and the Manoff Group (1,5,6,51,56-60). Thus, mothers/caretakers with children below two years will be interviewed about their socio-economic situation, food security, mothers' and children's food intake, care, time availability, access to health, water and sanitation, motor milestones, and access to FAO food security activities by trained enumerators. Anthropometric measurements will be taken from mothers, their children below two years of age, and, if possible from fathers, by a nutritionist especially trained in anthropometric measurements. Capillary blood samples will be taken from the surveyed children below two years to assess the micronutrient status (retinol binding protein (RBP), transferring receptor (TfR), hemoglobin as well as the morbidity status (C-reactive protein (CRP), acyl glycoprotein (AGP)) by medical trained research staff.

6.8.1 Interview

After written consent (see consent form in annex) the caretaker/mother of the child will be interviewed face-to-face by trained enumerators. Depending on the given infrastructure in the villages, participants are either invited in advance to come to a central meeting point, e.g. community center or primary school, to do the interview, or enumerators are coming to the homestead of the participants. If the interviews are conducted centrally in a public place, privacy will be assured by keeping an adequate distance between the interviewed participants that only the enumerators will hear the answers. In case village lists are not available, and systematic sampling is not possible, the EPI method will be used (51). Participants will then be invited on the day of selection to come to central meeting point at a certain time to avoid any inconvenience by waiting.

6.8.2 Anthropometric measurements

A central weighing and measuring station will be installed in every village. After the interview, mothers/caretakers (if possible the father as well) and their children will be send to the weighing and measuring station. Weight and height of children and adults will be measured according to an anthropometric protocol based on the WHO report "Physical status: the use and interpretation of anthropometry" (1995) (61).

Weight of children will be determined with the child wearing no clothing. Adults' weights will be taken while wearing light indoor clothing and no shoes. Heights and weights will be assessed to the nearest 0.5 cm and 0.1 kg, respectively. To determine the nutritional status of pregnant women, mid-upper arm circumference (MUAC) will be measured. All measures will be taken twice and the mean value is used for analysis (see ISAK manual (62)).

Anthropometric measurements will be taken with standardized equipment from Seca (Seca Gmbh & Co KG, Hamburg, Germany). Weight will be measured using standardized digital flatscales (Seca 874, capacity: 200 kg) with mother/child function.

Infants and small children are weighed while being held by the mother. The weight of the mother is assessed separately. Then the Mother-Child function ascertains the tare of the weight. Recumbent length will be taken from children with measuring boards (Seca 417, measurement range: 10–100 cm). The height of adults will be measured with a stadiometer (Seca 213, measuring range: 20 – 205 cm). A non-stretchable measuring tape will be used to take MUAC (Unicef).

After the weight and length assessment the children will be tested for edema. Edema will be diagnosed by applying moderate finger pressure on the tops of the child's feet. If there is edema, an impression will clearly remain for at least a few seconds (on both feet). Edema will be recorded as absent, mild (both feet/ankles), moderate (both feet/ankles plus lower legs, hands or lower arms) and severe (generalized edema including both feet, legs, hands, arms, and face) (51).

Two pairs of trained research staff, each consisting of a measurer and an assistant, will take all measurements. Privacy of the participants while taking their measurements will be assured.

6.8.3 Date of birth

Children's dates of birth (age) will be recorded either from health cards or health passport booklets. If no documents are available and the mother/caretaker doesn't know the child's birthdate, the age will be estimated based on the FAO Guidelines for Estimating the Month and Year of Birth of Young Children (FAO 2008) (63).

6.8.4 Blood samples

Blood samples will be taken at the central weighing and measuring station. A staff member from the Malawian Community Health Sciences Unit (CHSU) (waiting for confirmation) will obtain the blood sample for the biochemical parameters via a finger prick using sterile disposable micro lancets to obtain capillary blood.

The analysis of biochemical measurement will include assessment of retinol binding protein (RBP), hemoglobin, transferrin receptor (TfR), acyl glycoprotein (AGP) and C-reactive protein (CRP).

RBP, TfR, AGP and CRP will be analyzed using sandwich ELISA technology (64). With this technology it is possible to combine the measurements of the four proteins (TfR, RBP, CRP, AGP). Instead of using four different ELISA methods with different chemicals and procedures only one method is used. This also reduces the necessary amount of blood. A plasma volume of 2 times 7.5 µl is sufficient to do a double measurement of all four proteins. Directly after taking the blood samples they will be centrifuged and

serum/plasma is stored in 0.2 mL PCR tubes. These tubes can also be directly used in an automatic pipettor to avoid the tedious and error prone manual pipetting. Samples will be stored on ice in a high efficient styrofoam box with more than 5 cm thick walls and tightly closing lid. All blood samples will be stored on ice until the end of the survey. Analysis of blood samples will be done by Dr. Jürgen Erhardt, DBS-Tech, Germany.

Hemoglobin concentration will be assessed immediately at the field site with a portable HemoCue Hb 201* analyzer (HemoCue, Grossostheim-Germany) following operating guidelines (Hemocue Hb 201+ operating manual. HemoCue GmbH, Grossostheim, Germany). The analyzer will be calibrated with appropriate control solutions before starting measurements every time.

6.8.5 Motor milestones

Motor milestones will be assessed according to the Motor Development Study component of the WHO Multicenter Growth Reference Study (MGRS)(65). The following six distinct gross motor milestones will be recorded: sitting without support, hands-and knees crawling, standing with assistance, walking with assistance, standing alone, and walking alone. To assess all milestones, the standardized testing procedures from MGRS will be adopted. A milestone will only be considered as achived, if all given criteria are met.

6.8.6 Data collected on TIPs and Intervention

Several qualitative data collection methods will be used to observe and evaluate the TIPs process. The whole TIPs process will be observed by the PhD students. Before the TIPs starts, the observers will hold a meeting and agree on a structured guideline how and what will be recorded during the three TIPs steps. Village background information will as well be obtained from FICA and other secondary sources.

During the intervention focus group discussions as well as open one-on-one interviews will be held with family members (especially women and elderly), extension workers and nutritionists. Focus group discussions and one-on-one interviews are qualitative empirical research methods and imply that questions are posed in a colloquial manner instead of a standardized questionnaire. The focus group discussions will follow a general interview guide approach. During the focus group discussion participants are encouraged to talk freely about the subject. This will enlarge the understanding of how behavior change messages have been integrated in daily life, how behavior has changed and will as well identify obstacles to the implementation. Therefore, focus group discussions will allow gaining deeper understanding of the complex research background. (66-68).

6.9 Quality assurance

6.9.1 Statement of Compliance

The study will be conducted in accordance with the design and specific provisions of this Institutional Review Board (IRB) approved protocol. Good Epidemiological Practice (GEP) as recommended in the "Guidelines for proper conduct of epidemiological research" provided by the International Epidemiological Association (IEA).

The principal investigators will assure that no deviation from or changes to the protocol will take place without prior agreement from the sponsor and documented approval from the IRB. The principal investigator will promptly report to the IRB and the sponsor any changes in research activity and all unanticipated problems.

6.9.2 Translation of Questionnaire

Questionnaires will be designed in English and translated into Chichewa and Tumbuka by native speaking nutritionists. Ms. Gabriela Chapota, lecturer in Nutrition at the National Resources College, will be responsible for the translation from English into Tumbuka, the local language of Mzimba study site. Mr. Numeri Geresomo, lecturer in Human Nutrition at Bunda College, will be in charge to translate the questionnaire from English into Chichewa, the local language in Kasungu district and the official language of Malawi. The translated questionnaires will be tested on 3 to 5 native speakers. To assure analogous translations, independent, native speaking nutritionists will translate both versions of the questionnaire back into English.

6.9.3 Recruitment of field staff

Dr. Beatrice Mtimuni from Bunda College has wide-ranging experience in nutrition related field research. Therefore, Dr. Mtimuni will be responsible to contact and recruit experienced enumerators. Main requirements for enumerators are:

- language skills (Chichewa and Tumbuka)
- minimum of knowledge in nutrition related research and basic interview experiences

A total of 12 enumerators will be enrolled in the data collection process.

2 medical trained research staff will be recruited from CHSU to take blood samples and to assist the HemoCue analysis.

6.9.4 Training and monitoring of data collectors

Enumerators: The enumerators will be trained on the questionnaire. A guideline on how to conduct the interview will be developed and used during the training workshop. Interviews will be conducted pairwise. One enumerator will ask the questions, the other one will record the answers. The training will as well include the correct assessment of motor milestones based on the observation criteria used in the WHO MGRS (65).

During the data collection process enumerator teams will be matched randomly every day. Interviewers will be systematically and frequently monitored and completed questionnaires will be controlled for missing data and consistency on a daily basis.

Anthropometric measurements: In order to assure that all measurements are taken in the same way the training for taking anthropometric measurements will be based on the WHO Child Growth Standards "Training Course on Child Growth Assessment" (69). Ms. Gabriella Chapota and Dr. Beatrice Mtimuni will carry out the training. Furthermore the training curricula will cover issues of sensitivity to local customs, dress, and practices of modesty. Correct handling of equipment will as well be included in the training.

To avoid problems in this area, observers of the same sex as the subjects will be employed. Since the research project mainly focuses on young children and their mothers/caretakers, women will be recruited to take the measurements. Survey leaders will frequently visit the weighing and measuring station. These visits will serve to verify that the anthropometry protocol is being implemented properly and consistently. Retraining sessions will be arranged when a lack of standardization is observed among the researchers.

6.9.5 Assurance of communication

Supervisors are responsible for the technical quality of the surveys for which the survey teams under their charge are conducting. Enumerators will be randomly assigned to a supervisor. One supervisor will be in charge of three survey teams. The duties of a supervisor include advice and control of the accuracy of the survey data.

The research team will include a translator for the research supervisors from Germany. This will assure the communication between all research team members as well as participants. Survey leaders and survey supervisors will record all important points in a notebook as soon as possible, including observations, ideas, problems, actions taken to address these problems, and the reasoning behind any decisions taken.

Survey leaders, supervisors, and surveyors will meet daily after the survey implementation.

6.9.6 Pretest

A pretest of the entire data collection process will be conducted in villages not selected as clusters for the baseline survey. The pretest serves to ensure that the questionnaire is fully understood by the enumerators as well as to test the enumerators' behaviour in conducting the interviews. The results will be evaluated by the trainers and a final consultation is held with the enumerators. Difficulties encountered by the enumerators with the questionnaire will be discussed and eventually phrasing or translations will be adjusted. The aim is to clarify any remaining uncertain points and to reach an agreement on the final questionnaire to be used.

6.9.7 Registration of study

The IMCF research study will be registered at the German Clinical Trials Register (DRKS). The DRKS is an open access online register for clinical trials, which allows all users to search, register and share information on clinical trials. The DRKS is free and publicly accessible. The DRKS is an approved Primary Register in the WHO network since October 2008 and thus meets the requirements of the International Committee of Medical Journal Editors (ICMJE).

6.10 Data protection

Data management procedures will protect confidentiality of all data collected on individuals.

All Investigators and study site staff involved with this study must comply with the requirements of the respective data protection laws in Malawi and Germany with regard to the collection, storage, processing and disclosure of personal information. Access to collated participant data will be restricted to the survey management and stored in a locked cupboard.

Each subject will be assigned a unique identification code that will be used for data entry and analysis. To safeguard confidentiality, subject records are accessible only to the team doing the initial data entry, and the individual checking as a part of the dual entry system. Computers used to collate the data will have limited access measures via user names and passwords. Identity information and consent forms are not kept in the main computerized data file, but in a hard copy kept in a locked cabinet available only to the principle investigators. The front page of the individual record with consent and individual identity information is separated from the rest of the record, which contains only the individual code number.

Test tubes and specimens used by laboratory staff will be labeled by individual code numbers only. The collected blood samples will only be used for the specific purpose covered by the informed consent given.

Published results will not contain any personal data that could allow identification of individual participants.

7 Dissemination of findings

Reports on the project's progress and regular monitoring of the project activities will be provided by the FAO consultant, the Giessen PostDoc and the Principal Investigators. The research project team will be responsible to produce six-monthly progress reports, which will

- contain information on main activities and compliance with the work plan;
- identify any problems and constrains encountered during the research progress,
- provide recommendations for corrective measures;
- if necessary, revise the work plan for the following reporting period.

A contact information database will be created and maintained that will be used for group/individual mailings of paper documents and to facilitate telephone and fax communications between the Project Management, Site Management, and TAC. The project visibility will be enhanced by the launching of a FAO project website. The website will be updated at least on a 6 monthly basis.

Lessons learnt from the project and research results will be shared through:

- participation and presentations in relevant conferences and technical consultations regarding nutrition and feeding of infants and young children,
- preparation of research articles to be submitted to scientific journals
- documentation of case studies
- Preparation of guidelines and technical recommendations on improved complementary feeding using local resources.

8 Personnel

Sponsor/Donor	Food and Agriculture Organization of the United Nations (FAO)
Principal investigator	JLU-Giessen: Prof. Dr. MB Krawinkel, Ms I Jordan<br>Bunda College of Agriculture: Dr. B Mtimuni,
Co-Investigators	Ms G Chapota, Ms J Kuchenbecker
Management Committee	FAO: Ms E Mühlhoff, Ms G Kennedy<br>JLU-Giessen: Prof. Dr. MB Krawinkel, I Jordan<br>Bunda College of Agriculture: Dr. B. Mtimuni
Project team<br>- survey leader<br>- survey supervisor	 I Jordan and Dr. B Mtimuni<br>Ms G Chapota and Ms J Kuchenbecker
Technical Advisory Committee	

The research has been developed and will be carried out by the Institute of Nutritional Sciences, Justus Liebig University Giessen, Germany in collaboration with Bunda College, Lilongwe, Malawi. A PhD student from Bunda College (Gabriela Chapota) and a PhD student from JLU-Giessen (Judith Kuchenbecker) will undertake data collection and analysis; they will be supported by MSc students from Germany (e.g. Ms Leonie Hoeber from May to December 2011) and Malawi (e.g. Ms Ethel Luhanga).

Representatives from FAO (e.g. Gina Kennedy, consultant), Bunda College of Agriculture (Dr. Beatrice Mtimuni) and JLU-Giessen (Irmgard Jordan, PostDoc to-be) will be involved in project management and technical implementation. They will provide oversight of the project's implementation, approve workplans and associate budgets, and decide upon adjustments to the project implementation strategy as required. Furthermore, they will be responsible for ensuring that information is effectively shared between FAO, Giessen and national research institutes and governments, and between country-level teams and FAO headquarters and JLU Giessen in Germany.

A Technical Advisory Committee (TAC) (members to be confirmed) has been formed including researchers and practitioners from academic institutions, UN partner agencies and NGOs having been involved in similar research and/or field work on complementary feeding, behavior change and nutritional impact studies. The TAC will provide advice on the research methodology, preliminary research results and other technical issues as they arise.

At country level, the official collaboration between IMCF research group (JLU and Bunda College) and FAO FICA project will start with a common meetings on national and district level organized by FAO in May (between 9th and 22nd) in Kasungu, Malawi. Relevant government departments and district administration/representatives will be invited. A second meeting round will be held as soon as the results of the nutrition survey are available and the cluster randomization needs to be conducted.

9 References

1. PAHO. WHO | Guiding principles for complementary feeding of the breastfed child [Internet]. 2003 [cited 2011 Apr 13];Available from: http://www.who.int/child_adolescent_health/documents/a85622/en/index.html
2. Dirorimwe C. Healthy Food, Happy Baby, LivelyFamily - Improved Feeding Practices and Recipes for Afghan Children and Mothers [Internet]. 2008;Available from: ftp://ftp.fao.org/docrep/fao/011/ak183e/ak183e.pdf
3. Dirorimwe C. Improved Complementary Foods Recipe Booklet Family Foods for Breastfed Children in Zambia [Internet]. 2007 [cited 2011 Apr 13];Available from: ftp://ftp.fao.org/docrep/fao/010/ai208e/ai208e.pdf
4. Dickin K, Griffiths M, Piwoz E. Designing by dialogue. A Program Planner's Guide to Consultative Research for Improving Young Child Feeding. Washington, DC: Academy for Educational Development. 1997;
5. Manoff Group. Trials of Improved Practices (TIPs)Giving Participants a Voice in Program DesignTrials [Internet]. 2005 [cited 2011 Apr 18];Available from: http://www.manoffgroup.com/resources/summarytips.pdf
6. Dirorimwe C. TIPs guidelines. PART II Guiding notes for TIPs trainers (unpublished data). 2010;
7. The Malawi Vulnerability Assessment Committee Bulletin [Internet]. 2010 [cited 2011 Apr 13];Available from: http://www.unmalawi.org/reports/Sit_reports/MVAC%20Report_June%202010.pdf
8. FEWSNET. Malawi Food Security Outlook [Internet]. [cited 2011 Apr 14];Available from: http://www.fews.net/pages/country.aspx?gb=mw
9. Daelmans B, Mangasaryan N, Martines J, Saadeh R, Casanovas C, Arabi M, et al. Strengthening action to improve feeding of infants and young children 6 to 23 months of age: summary of a recent World Health Organization/UNICEF technical meeting, Geneva, 6-9 October 2008. In: WHO/UNICEF technical meeting on" Strengthening action to improve feeding of infants and young children 6-23 months of age in nutrition and child health programmes", Geneva, Switzerland, 6-9 October 2008. 2009. p. S236–S238.
10. National Staistical Office. Preliminary Report of the Malawi Demographic and Health Survey 2010 [Internet]. 2010;Available from: http://www.nso.malawi.net/images/stories/data_on_line/demography/MDHS2010/MDHS 2010MalawiPreliminaryReport2-28-11LTR.pdf
11. Zomba M, Macro ORC. Malawi-Demographic and Health Survey. 2004;
12. WHO. WHO | WHO Child Growth Standards: Methods and development: Length/height-for-age, weight-for-age, weight-for-length, weight-for-height and body mass index-for-age. [Internet]. [cited 2011 Apr 18];Available from: http://www.who.int/childgrowth/publications/technical_report_pub/en/index.html
13. UNICEF. Malawi - Statistics [Internet]. 2010 [cited 2011 Apr 14];Available from: http://www.unicef.org/infobycountry/malawi_statistics.html#76
14. Shet A, Mehta S, Rajagopalan N, Dinakar C, Ramesh E, Samuel N, et al. Anemia and growth failure among HIV-infected children in India: a retrospective analysis. BMC Pediatr. 2009;9(1):37.
15. Nalwoga A, Maher D, Todd J, Karabarinde A, Biraro S, Grosskurth H. Nutritional status of

children living in a community with high HIV prevalence in rural Uganda: a cross-sectional population-based survey. Tropical Medicine & International Health [Internet]. 2010 [cited 2011 Apr 12];Available from: http://doi.wiley.com/10.1111/j.1365-3156.2010.02476.x
16. USAID. HIV/AIDS Health Profile Malawi [Internet]. 2010;Available from: http://www.usaid.gov/our_work/global_health/aids/Countries/africa/malawi_profile.pdf
17. FAO/FICA. Food and Nutrition security programme: Improving food security and nutrition policies and programme outreach. Nutrition Baseline Survey in Kasungu and Mzimba. Unpublished report. 2008.
18. I-Life. Complementary foods recipe book for malawian children. 2010;
19. Motarjemi Y, K\äferstein F, Moy G, Quevedo F. Contaminated weaning food: a major risk factor for diarrhoea and associated malnutrition. Bulletin of the World Health Organization. 1993;71(1):79.
20. Brown K, Dewey K, Allen L. Complementary feeding of young children in developing countries: a review of current scientific knowledge. Geneva: World Health Organization; 1998.
21. Boyce DG, Lewis MR, Worm B. 6th report on the world nutrition situation: PROGRESS IN NUTRITION. Nature. 2010;466(7306):591–596.
22. Jonsson U. Strategy for Improved Nutrition of Children and Women in Developing Countries. A UNICEF Policy Review 1990-1. 1981;
23. Krawinkel MB. The value of Asian-Africa collaboration in food and health security. Asia Pac J Clin Nutr. 2009;18(4):570–576.
24. Keding GB. Linking nutrition security and agrobiodiversity: the importance of traditional vegetables for nutritional health of women in rural Tanzania. 2010;
25. Engesveen K, Shrimpton R. Nutrition education in the context of the United Nations Standing Committee on Nutrition activities and publications, 1985-2006. J Nutr Educ Behav. 2007 Dec;39(6):351-356.
26. Ruel MT. Can food-based strategies help reduce vitamin A and iron deficiencies? a review of recent evidence. International Food Policy Research Institute (IFPRI). 2001;(5).
27. Bhutta ZA, Ahmed T, Black RE, Cousens S, Dewey K, Giugliani E, et al. What works? Interventions for maternal and child undernutrition and survival. The Lancet. 2008;371(9610):417-440.
28. Ashworth A, Ferguson E. Dietary counseling in the management of moderate malnourishment in children. Food Nutr Bull. 2009 Sep;30(3 Suppl):S405-433.
29. Shi L, Zhang J. Recent Evidence of the Effectiveness of Educational Interventions for Improving Complementary Feeding Practices in Developing Countries [Internet]. 2010 [cited 2011 Apr 12];Available from: http://tropej.oxfordjournals.org/content/early/2010/06/16/tropej.fmq053.full.pdf+html
30. Victora CG, de Onis M, Hallal PC, Blossner M, Shrimpton R. Worldwide Timing of Growth Faltering: Revisiting Implications for Interventions. PEDIATRICS. 2010;125(3):e473-e480.
31. Gibson RS, Abebe Y, Hambidge KM, Arbide I, Teshome A, Stoecker BJ. Inadequate feeding practices and impaired growth among children from subsistence farming households in Sidama, Southern Ethiopia. Matern Child Nutr. 2009 Jul;5(3):260-275.
32. Wu L, Katz J, Mullany LC, Haytmanek E, Khatry SK, Darmstadt GL, et al. Association between Nutritional Status and Positive Childhood Disability Screening Using the Ten Questions Plus

Tool in Sarlahi, Nepal. Journal of Health, Population, and Nutrition. 2010;28(6):585.
33. Krebs NF, Hambidge KM, Mazariegos M, Westcott JL, Goco N, Wright LL, et al. Complementary feeding: a Global Network cluster randomized controlled trial. BMC Pediatr. 2011;11(1):4.
34. Kwena AM, Terlouw DJ, De Vlas SJ, Phillips-Howard PA, Hawley WA, Friedmann JF, et al. PREVALENCE AND SEVERITY OF MALNUTRITION IN PRE-SCHOOL CHILDREN IN A RURAL AREA OF WESTERN KENYA -- KWENA et al. 68 (4 Supplement): 94 -- American Journal of Tropical Medicine and Hygiene [Internet]. 2003 [cited 2011 Apr 12];Available from: http://www.ajtmh.org/cgi/reprint/68/4_suppl/94
35. Phuka JC, Maleta K, Thakwalakwa C, Cheung YB, Briend A, Manary MJ, et al. Complementary Feeding With Fortified Spread and Incidence of Severe Stunting in 6- to 18-Month-Old Rural Malawians. Arch Pediatr Adolesc Med. 2008 Jul 1;162(7):619-626.
36. Lin CA, Manary MJ, Maleta K, Briend A, Ashorn P. An Energy-Dense Complementary Food Is Associated with a Modest Increase in Weight Gain When Compared with a Fortified Porridge in Malawian Children Aged 6–18 Months. The Journal of Nutrition. 2008 Mar 1;138(3):593 -598.
37. Owino VO, Amadi B, Sinkala M, Filteau S, Tomkins A. Complementary feeding practices and nutrient intake from habitual complementary foods of infants and children aged 6–18 months old in Lusaka, Zambia. African Journal of Food, Agriculture, Nutrition and Development. 2008;8(1).
38. Adu-Afarwuah S, Lartey A, Brown KH, Zlotkin S, Briend A, Dewey KG. Home fortification of complementary foods with micronutrient supplements is well accepted and has positive effects on infant iron status in Ghana. The American Journal of Clinical Nutrition. 2008 Apr 1;87(4):929 -938.
39. Ouédraogo HZ, Nikièma L, Somé I, Sakandé J, Dramaix-Wilmet M, Donnen P. Home-based practices of complementary foods improvement are associated with better height-for-age Z score in 12-23 months-old children from a rural district of Burkina Faso. African Journal of Food, Agriculture, Nutrition and Development. 2008;8(2):204–218.
40. Guyon AB, Quinn VJ, Hainsworth M, Ravonimanantsoa P, Ravelojoana V, Rambeloson Z, et al. Implementing an integrated nutrition package at large scale in Madagascar: The Essential Nutrition Actions Framework. Food &# 38; Nutrition Bulletin. 2009;30(3):233–244.
41. Phuka JC, Maleta K, Thakwalakwa C, Cheung YB, Briend A, Manary MJ, et al. Postintervention growth of Malawian children who received 12-mo dietary complementation with a lipid-based nutrient supplement or maize-soy flour. The American Journal of Clinical Nutrition. 2009 Jan 1;89(1):382 -390.
42. Ramakrishnan U, Nguyen P, Martorell R. Effects of micronutrients on growth of children under 5 y of age: meta-analyses of single and multiple nutrient interventions. American Journal of Clinical Nutrition. 2008;89(1):191-203.
43. Defourny I, Minetti A, Harczi G, Doyon S, Shepherd S, Tectonidis M, et al. A Large-Scale Distribution of Milk-Based Fortified Spreads: Evidence for a New Approach in Regions with High Burden of Acute Malnutrition. PLoS ONE. 2009;4(5):e5455.
44. Macharia-Mutie CW, Brouwer ID, Mwangi AM, Kok FJ. Complementary feeding practices and dietary intake among children 12-23 months in Mwingi district, Kenya. 2010 [cited

2011 Apr 12];Available from: http://inderscience.metapress.com/app/home/contribution.asp?referrer=parent&backto=issue,5,7;journal,1,5;linkingpublicationresults,1:121116,1
45. Chhagan MK, Van den Broeck J, Luabeya KKA, Mpontshane N, Tomkins A, Bennish ML. Effect on longitudinal growth and anemia of zinc or multiple micronutrients added to vitamin A: a randomized controlled trial in children aged 6-24 months. BMC Public Health. 2010;10(1):145.
46. Berti PR, Mildon A, Siekmans K, Main B, MacDonald C. An adequacy evaluation of a 10-year, four-country nutrition and health programme. International Journal of Epidemiology. 2010 Apr 1;39(2):613 -629.
47. Flax VL, Ashorn U, Phuka J, Maleta K, Manary MJ, Ashorn P. Feeding patterns of underweight children in rural Malawi given supplementary fortified spread at home. Maternal & Child Nutrition. 2007;4(1):65-73.
48. Flax VL, Thakwalakwa C, Phuka J, Ashorn U, Cheung YB, Maleta K, et al. Malawian mothers' attitudes towards the use of two supplementary foods for moderately malnourished children. Appetite. 2009;53(2):195–202.
49. Donegan S, Maluccio JA, Myers CK, Menon P, Ruel MT, Habicht JP. Two Food-Assisted Maternal and Child Health Nutrition Programs Helped Mitigate the Impact of Economic Hardship on Child Stunting in Haiti. The Journal of nutrition. 2010;140(6):1139.
50. Paul KH, Muti M, Chasekwa B, Mbuya MNN, Madzima RC, Humphrey JH, et al. Complementary feeding messages that target cultural barriers enhance both the use of lipid-based nutrient supplements and underlying feeding practices to improve infant diets in rural Zimbabwe. Maternal & Child Nutrition. 2010;:no-no.
51. Golden M, Brennen M, Brennan R. Measuring Mortality, Nutritional Status, and Food Security in Crisis Situations: SMART METHODOLOGY [Internet]. 2006;Available from: http://www.smartmethodology.org/images/stories/SMART_Methodology_08-07-2006.pdf
52. Hayes R, Moulton LH. Cluster randomised trials. Boca Raton: CRC Press; 2009.
53. RSeek.org R-project Search Engine [Internet]. 2011 Apr 18 [cited 2011 Apr 18];Available from: http://www.rseek.org/?cx=010923144343702598753%3Aboaz1reyxd4&newwindow=1&q=experiment&sa=Search+functions%2C+lists%2C+and+more&cof=FORID%3A11&siteurl=rseek.org%2F#1022
54. Mayring P. Qualitative Inhaltsanalyse Grundlagen und Techniken. 11th ed. Weinheim: Beltz; 2010.
55. MAXQDA - The Art of Text Analysis [Internet]. [cited 2011 May 5];Available from: http://www.maxqda.de/
56. WHO | Assessing the iron status of populations-second edition [Internet]. 2004 [cited 2011 Apr 14];Available from: http://www.who.int/nutrition/publications/micronutrients/anaemia_iron_deficiency/9789241596107/en/index.html
57. WHO-Indicators for assessing infant and young child feeding practices: Part II Measurement [Internet]. 2010;Available from: http://www.who.int/nutrition/publications/infantfeeding/9789241599290/en/index.html
58. Gross R, Kielmann A, Korte R, Schoeneberger H, Schultink W. Nutrition baseline surveys in

communities. Nutrition surveys and calculations: guidelines, software and additional information (Erhardt J, Gross R, eds.). Version current. 1997;
59. Deitchler M, Bellard T, Swindale A, Coates J. Validation of a measure of household hunger for cross-cultural use. 2010;Available from: http://pdf.usaid.gov/pdf_docs/PNADU857.pdf
60. FAO, Fanta. Guidelines for measuring household and individual dietary diversity [Internet]. 2008 [cited 2011 May 4];Available from: http://www.foodsec.org/fileadmin/user_upload/eufao-fsi4dm/docs/guidelines_MeasuringHousehold.pdf
61. WHO Expert Committee on Physical Status: the Use and Interpretation of Anthropometry. Physical status : the use and interpretation of anthropometry : report of a WHO Expert Committee. Geneva: World Health Organization; 1995.
62. Marfell-Jones M, Olds T, Stewart A, Carter L. International standards for anthropometric assessment. Potchefstroom, South Africa: ISAK; 2006.
63. FAO, IFAD. Guidelines for Estimating the Month and Year of Birth of Young Children [Internet]. 2008 [cited 2011 Apr 14]. Available from: ftp://ftp.fao.org/docrep/fao/011/aj984e/aj984e00.pdf
64. Erhardt JG, Estes JE, Pfeiffer CM, Biesalski HK, Craft NE. Nutritional Methodology. 2004;
65. Wijnhoven TMA, de Onis M, Onyango AW, Wang T, Bjoerneboe G-EA. Assessment of gross motor development in the WHO Multicentre Growth Reference Study.pdf. 2004 [cited 2011 Apr 18];Available from: http://www.who.int/childgrowth/mgrs/en/fnb_motor_37_45.pdf
66. Bortz J, Doering N. Forschungsmethoden und Evaluation : für Human- und Sozialwissenschaftler   mit 87 Tabellen. 4th ed. Heidelberg: Springer-Medizin-Verl.; 2009.
67. Greenbaum T. Moderating focus groups : a practical guide for group facilitation. Thousand Oaks Calif.: Sage Publications; 2000.
68. Krueger R. Focus groups : a practical guide for applied research. 3rd ed. Thousand Oaks Calif.: Sage Publications; 2000.
69. WHO. Training course on child growth assessment. 2008 Geneva;
70. Research Tools [Internet]. 2011 Apr 18 [cited 2011 Apr 18];Available from: http://www.wtgrantfdn.org/resources/overview/research_tools/research_tools

10 Annexes

10.1 LOGICAL FRAMEWORK OF RESEARCH PROJECT

Project Objectives/Research Objective	Indicators	Means of Verification	Assumptions/Risks
**Project Impact:**<br>Child nutritional status is improved<br><br>**Research objective:**<br>Evaluate the impact of the project on child nutritional status in FAO food security projects	**Key Impact Indicators** –:<br>a. Improved child nutritional status:<br>b. Reduced Stunting<br>c. Reduced Anemia<br>d. Reduced vit A deficiency	Baseline and Impact surveys<br>of children 0-23 months in FAO food security project and non-project areas	Households are willing to participate in all aspects of data collection
**Outcome:**<br>Complementary feeding (CF) practices and dietary intakes of children 0-23 months are improved<br><br>**Research objective:**<br>Assess changes in CF practices across intervention and control households	a. Improved CF practices (Selected WHO IYCF)<br>b. Decreased prevalence of anemia, vit A deficiency<br>c. Increased diversity of foods fed to children 6-23 months<br>d. Increased frequency of feeding semi-solid/solid foods<br>e. Mothers's behaviour score	Two levels of assessment<br>(TIPs and Impact assessment)	The complementary feeding components are implemented according to plan and the TIPs correctly assesses the decision making processes at household level that lead to sustained changes in feeding practices
**Output 1:**<br><br>Knowledge and practices related to CF are improved	Knowledge and practices (breastfeeding, CF and safe handling/preparation of food, active feeding, frequency of feeding, quantity, quality) of key change agents for child feeding practices are improved.<br><br>Decreased prevalence of ARI and diarrhoea.	Two levels of assessment (TIPs and Impact assessment)	The complementary feeding components are implemented according to plan and the TIPs facilitators correctly assess the decision making processes at household level that lead to sustained changes in feeding practices

Table cont.

Project Objectives/Research Objective	Indicators	Means of Verification	Assumptions/Risks
**Project activity: Training of TIPs facilitators**	TIPs facilitators are trained		
**Research activities:**<br>TIPs consultants conducts the training of the TIPs facilitators according to the guidelines and ensures that TIPs facilitators have a common understanding of the food needs of family members most at risk of becoming malnourished and are familiarized on the approach to use when counseling the TIPs families.	a. TIPs training is conducted according to the guideline.<br>b. TIPs facilitators are tested whether they are familiar with nutrition education messages.<br>c. TIPs facilitators' capability conducting data assessment according to the TIPs guidelines is tested	TIPs training will be monitored and a pretest will be conducted and analyzed using the TIPs toolkit for TIPs implementation.	Participants at the training are willing to be observed during the training and willing to undergo a final knowledge assessment.
**Project activity: TIPs implementation**	Behaviour change messages and improved CF recipes developed and tested with mothers		
Research activities:<br>TIPs formative research generates appropriate behaviour change messages and CF recipes that are:<br><br>Seasonally appropriate,<br>nutritionally adequate,<br>culturally acceptable<br>affordable.	a. # of appropriate behaviour change messages developed<br>b. Assessment of the appropriateness, feasibility and acceptability of nutrition education messages<br>c. Assessment of the cultural acceptability of recipes<br>d. Assessment of the affordability of recipes<br>e. Assessment of factors affecting feeding practices and nutritional status<br>f. Assessment of facilitators/nutrition promoters' knowledge and skills in dietary counseling (in terms of accuracy of content and quality of the interaction)<br>g. Assessment of facilitators/nutrition promoters' knowledge and skills in facilitating cooking demonstrations	Research team monitoring TIPs<br><br>In-depth qualitative and quantitative information of TIPs and control HH<br><br>Cost effectiveness verified and fine tuned through Linear Programming tool.<br><br>Qualitative research (focus groups) on message acceptability, cultural acceptability of recipes and affordability of new recipes	Partnerships with FAO food security projects are established, funded and initiated at the same time as the research

Table cont.

**Project Objectives/Research Objective**	**Indicators**	**Means of Verification**	**Assumptions/Risks**
**Research activities**<br><br>The potential for meeting the nutritional requirements of children 6-23 months of age with locally available and affordable foods is assessed.	a. Assessment of the ability of local foods to meet nutrient requirements by season<br>b. % of nutrient requirements met by improved recipes<br>c. Calculation of energy and nutrient density of CF<br>d. Number of CF recipes that meet nutritional requirements by season<br>e. Assessment of the cost and affordability of improved local recipes	Seasonal calendars of food availability developed<br><br>Locally appropriate recipes developed<br><br>Nutrient content of CF recipes, including energy and micronutrient density assessed	Food composition data are available for local foods (local food varieties) or samples of local foods or recipes are collected for laboratory analysis
**Research activities**<br><br>Improvements in dietary intakes and child nutritional status	a. Assessment of dietary intakes<br>b. Assessment of child growth<br>c. Assessment of motormilestones<br>d. Biochemical assessment of micronutrient and morbidity status	Initial and follow-up assessments of diet, growth, motormilestones and collection of blood samples	No drop out or refusal to participate
**Project activity –dissemination of feeding recommendations and recipes**	Disseminate through local community groups messages and recipes developed through TIPs		
**Research Activities**<br><br>Evaluate the effectiveness of disseminating behaviour change messages and improved recipes for CF, in association with a food security intervention	a. Assess the process through which messages and recipes are promoted and taken up<br>b. Assess the factors that encourage or hinder uptake of recipes (strength of community organization, combination of FS interventions and CF interventions)<br>c. Assess the frequency and quality of nutrition education and dietary counselling with families and community groups<br>d. Assess the effectiveness of different delivery channels	Qualitative research (i.e. social mapping, focus groups discussion)<br><br>Survey assessments	Presence in communities of women's groups or appropriate extension agents. Messages are delivered to all persons within family/community that influence behaviours and practices

10.2 IMCF Malawi work plan

	2011												2012												2013											
	1	2	3	4	5	6	7	8	9	10	11	12	1	2	3	4	5	6	7	8	9	10	11	12	1	2	3	4	5	6	7	8	9	10	11	12
Literature review	X	X	X	X																																
Final selection of academic institution in Malawi	X	X																																		
Staff recruitment PhD in Germany and Malawi	X	X	X	X																																
Institutional and implementing arrangements	X	X	X	X																																
Constitute TAC			X																																	
Refined research design	X	X	X	X	X																															
Translation of questionnaires				X	X																															
Application for ethical clearance					X																															
Set up an office in Kasungu					X																															
Contact representatives of local institutions and organizations					X																															
Collection of structural data					X	X																														
Preparation of training program for data collectors					X	X																														
Staff recruitment (field)					X																															
Kick off workshop Kasungu?					?	?																														
Staff training						X																														
Pretest							X																													
**Baseline survey: data collection**							X																													
Data entry							X																													
Initial analysis of the survey							X	X																												
Analysis of blood samples								X																												
Informing TIPs implementers and TAC of survey results								X																												
More detailed analysis and report								X	X																											
TIPs process monitoring & evaluation								X	X	X	X	X	X	X	X	X	X	X	X	X	X	X	X	X	X	X	X	X								
Refining questionnaire for impact assessment																													X	X						
Staff recruitment																														X						
Staff training																															X					
Pretest																															X					
**Impact assessment survey: data collection**																																X				
Data entry																																X				
Impact assessment analysis and report																																X	X	X	X	X
Analysis and research write-up and dissemination of publications																																		X	X	X

10.3 Particpants card

The Particpants card will be distributed to the participating households after informed consent.

BMI

< 18.5	Underweight
19 – 25	Normal
25 – 30	Overweight
> 30	Obese

WAZ HAZ (children under 2 years)

0	0	Normal
< -2	< -2	Moderate
< -3	< -3	Severe

Hemoglobin (Hb)
(children 6-59 months)

< 7	Severe anaemic
< 11	Anaemic
≥ 11	Normal

Participants Card

Name:

____

participated in the Baseline Survey of IMCF joined FAO – Justus Liebig University and Bunda College research study.

For further information please contact:

Dr. Beatrice Mtimuni
Home Economics and Human Nutrition
Bunda College of Agriculture
P.O. Box 219
Lilongwe, Malawi

Tel: 0888851870

Data assessed include:

Interview

Household and food security situation ☐

Child feeding practices ☐

Clinical part

Anthropometric measurements ☐

Test for edema (child) ☐

Interview on motor milestones of child ☐

Blood test for vitamin A and iron status ☐

Hemoglobin prick test ☐

Results:

Anthropometry, Hemoglobin and Edema

	Height (cm)	Weight (kg)	BMI
Mother			
Father			

	Age	Height (cm)	Weight (kg)	WAZ	HAZ	Hb (g/l)
Child 1						
Child 2						

	Edema
Child 1	
Child 2	

Conclusion

Nutritional status of mother alarming? no ☐ yes☐

Nutritional status of child alarming? no ☐ yes☐

→ If yes, please visit the nearest health post

10.4 Intra-class correlation and minimal detectable effect size

Reference: (70)

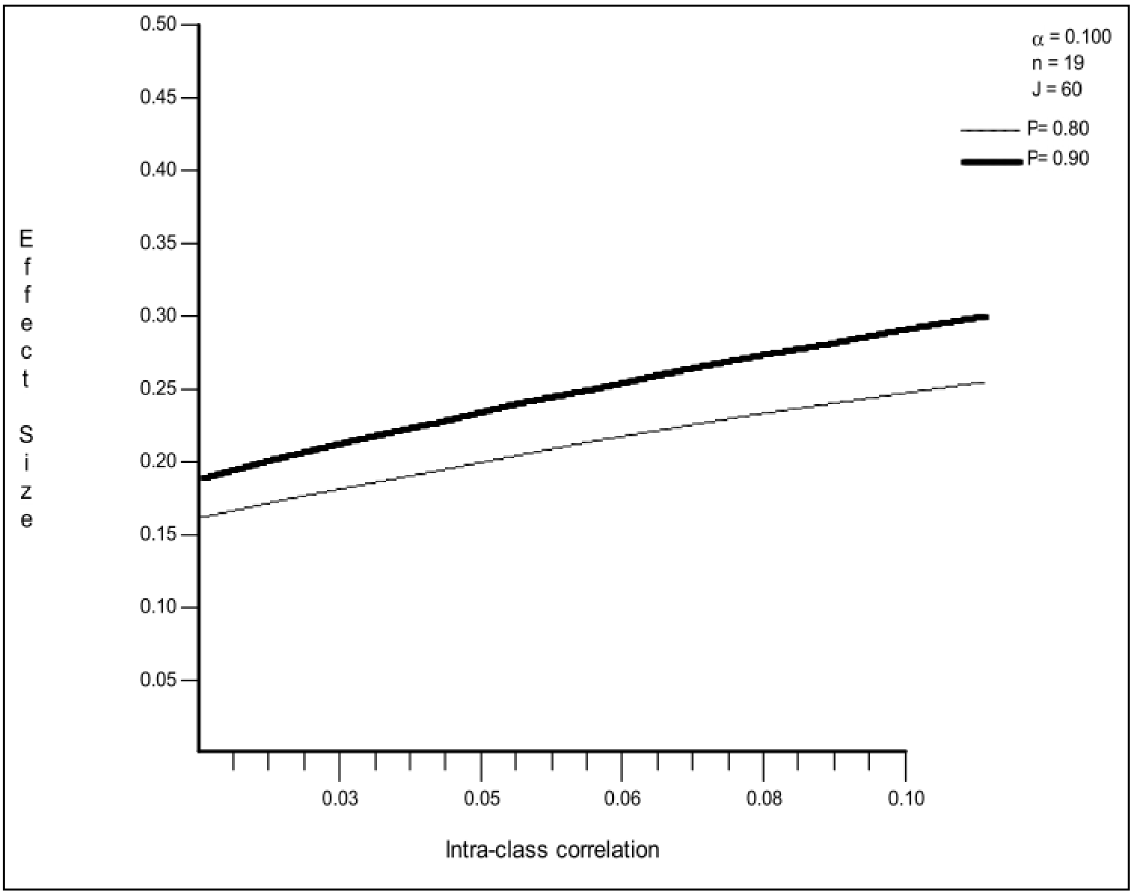

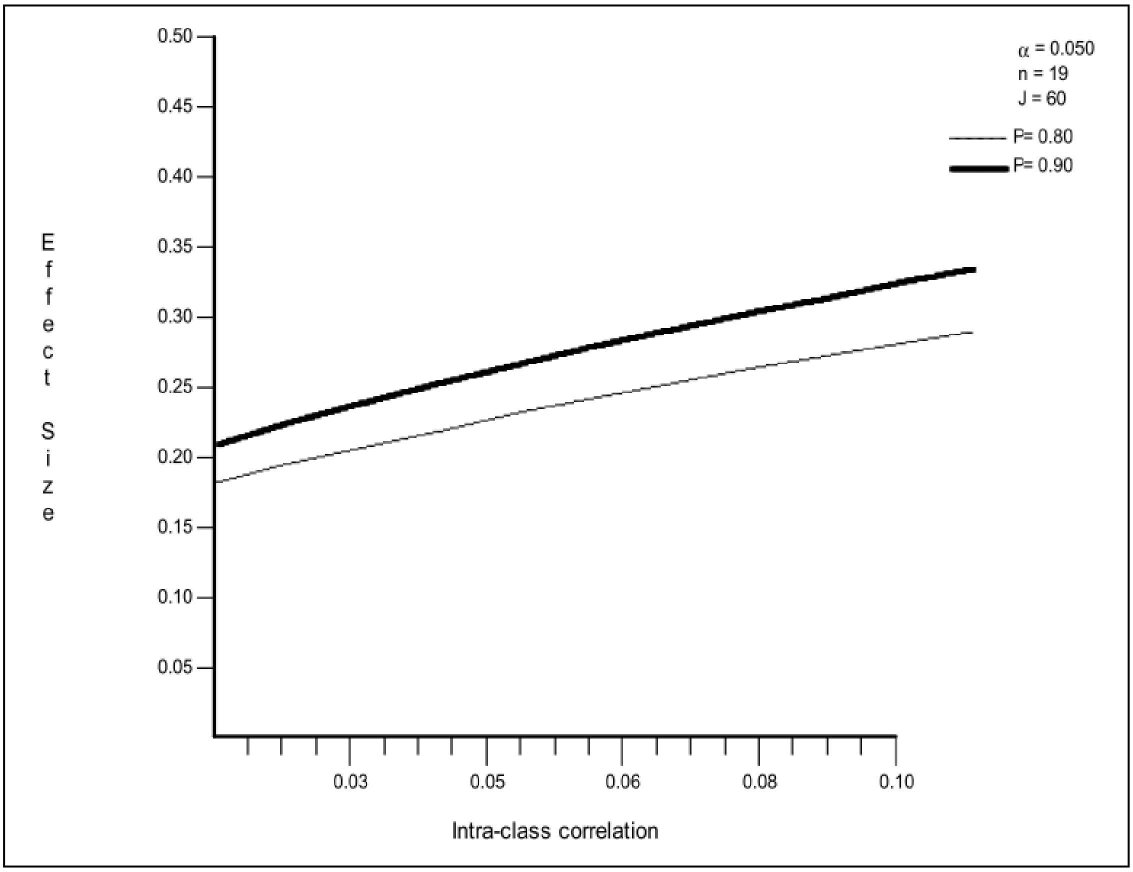

10.5 Estimated Budget (operational costs)

Component Description	year 1	year 2	year 3	total
Interviewers and translators	20,000.00	0.00	20,000.00	
**Sub-total staff**	**20,000.00**	**0.00**	**20,000.00**	**40,000.00**
Interviewer training	5,000.00	0.00	5,000.00	
**Sub-total training**	**5,000.00**	**0.00**	**5,000.00**	**10,000.00**
Local transport (lumpsum)	5,000.00	2,500.00	5,000.00	
**Sub-total travel**	**5,000.00**	**2,500.00**	**5,000.00**	**12,500.00**
Expendable equipment for surveys	3,700.00	0.00	3,700.00	
**Subtotal Expendable Equipment**	**3,700.00**	**0.00**	**3,700.00**	**7,400.00**
Technical instruments				
Office equipment				
**Subtotal Non Expendable Equipment**				
Ethical Commission fee (US$)	**150.00**			**150.00**
**Total US$**				**70,050.00**

Improving the dietary intakes and nutritional status of infants and young children through improved food security and complementary feeding counselling (IMCF)

Bunda College and Justus Liebig University Giessen in collaboration with FAO/ FICA

Questionnaire

Baseline Survey

Kasungu and Mzimba District

July 2011

Date of the Interview (month/ day/year)	|_|_| |_|_| |_|_|		DATE	
Interviewer Number 1:<br><br>Interviewer Number 2:		|_|_| |_|_|	INTNO1<br><br>INTNO2	
Identity Number of the household<br>(District: Kasungu = K, Mzimba = M,<br>EPA, No. of Village)	|_| District |_|_| EPA	|_|_|_|_| Village no	|_|_|_|_| Family	|_|_| IDNO Child

Identity No. ☐☐☐- ☐☐☐☐- ☐☐☐☐- ☐☐

Time of assessment: __ __ : __ __

Anthropometry of parents

Weight of the **mother** (in kg)	WM1: __ __ , __ kg<br>WM2: __ __ , __ kg	WEIGHTMO	__ __ , __ kg
Height of the **mother** (in cm)	HM1: __ __ __ , __ cm<br>HM2: __ __ __ , __ cm	HEIGHTMO	__ __ __ , __ cm
Weight of the **father** (in kg)	WF1: __ __ , __ kg<br>WF2: __ __ , __ kg	WEIGHTFA	__ __ , __ kg
Height of the **father** (in cm)	HF1: __ __ __ , __ cm<br>HF2: __ __ __ , __ cm	HEIGHTFA	__ __ __ , __ cm

Anthropometry of children below 2 years

	Youngest child under 2 = 1	Second youngest under 2 = 2		
Date of birth (day/month/year)<br>*Pls note: check birth date with health card; Use local calendar and stop interview if birth date cannot be identified for a specific month*	__ __ day __ __ month __ __ year	__ __ day __ __ month __ __ year	BIRTHDAT1<br>BIRTHDAT2	__ __ __ __ __ __<br>__ __ __ __ __ __
Weight of the children (in kg)	__ __ , __ kg<br>__ __ , __ kg	__ __ , __ kg<br>__ __ , __ kg	WEIGHTCH1<br>WEIGHTCH2	__ __ , __ __ kg<br>__ __ , __ __ kg
Length of the children (in cm)	__ __ __ , __ cm	__ __ __ , __ cm	HEIGHTCH1<br>HEIGHTCH2	__ __ __ , __ cm<br>__ __ __ , __ cm
Sex of child? 1= male 2= female	__	__	SEXCHILD1<br>SEXCHILD2	__<br>__

Hemoglobin of children below 2 years

Hemoglobin child 1 (g/dl)	└┴┘ g/dl	HEMCH1	
Hemoglobin child 2 (g/dl)	└┴┘ g/dl	HEMCH2	

Edema of children below 2 years

Edema child 1	1= yes<br>2= no	EDEMCH1	└┘
Edema child 2	1=yes<br>2= no	EDEMCH2	└┘

Motor milestones *(youngest child = 1; older child = 2)*

Sitting without support	Child's head is erect<br>Child does not use arms or hands to balance<br>Child sits up straight for at least 10 seconds	1= yes<br>2= no (inability)<br>3= no (refusal)<br>9= unable to test	MOTHSIT1<br>MOTOSIT2	└┘<br>└┘
Hands-and-knees crawling	Alternating movement forward or backward on hands & knees<br>Child's stomach does not touch the ground<br>Continuous and consecutive movements, at least 3 in a row	1= yes<br>2= no (inability)<br>3= no (refusal)<br>9= unable to test	MOTOC1<br>MOTOC2	└┘<br>└┘
Standing with assistance	Child is in an upright position on both feet<br>Child holds onto a stable object with both hands without leaning on it<br>Child's body does not touch the stable object<br>Child stands with assistance for at least 10 seconds	1= yes<br>2= no (inability)<br>3= no (refusal)<br>9= unable to test	MOTOST1<br>MOTOST2	└┘<br>└┘
Walking with assistance	Child is in an upright position with the back straight<br>Child makes sideways or forward steps by holding onto a stable object<br>One leg moves forward while the other supports part of the body weight<br>Child takes at least 5 steps in this manner	1= yes<br>2= no (inability)<br>3= no (refusal)<br>9= unable to test	MOTOWA1<br>MOTOWA2	└┘<br>└┘
Standing alone	Child is in an upright position on both feet with the back straight<br>Child stands alone for at least 10 seconds	1= yes<br>2= no (inability)<br>3= no (refusal)<br>9= unable to test	MOTOS1<br>MOTOS2	└┘<br>└┘
Walking alone	Child is in an upright position with the back straight<br>One leg moves forward while the other supports most of the body weight<br>Child takes at least 5 steps independently	1= yes<br>2= no (inability)<br>3= no (refusal)<br>9= unable to test	MOTOW1<br>MOTOW2	└┘<br>└┘

Household Questionnaire

Identity Number of the household	District EPA	Village no	Family

1	What is the sex of household head?	1= male<br>2= female	HEADHH	
2	What is the marital status of the household head	1= Currently Married – monogamous<br>2= Currently Married – polygamous<br>3= Widowed<br>4= Divorced<br>5= Single<br>6= Orphan (under 18 years of age)	HEADMAR	
3	What is the religion of the head of this household?	1= Catholic<br>2= CCAP<br>3= Anglican<br>4= Seventh Day Advent/Baptist<br>5= Other Christian<br>6= Muslim<br>7= Hindu<br>8= No Religion<br>99= Others (specify):	RELHHH	
4	What is your (HH) tribe or ethnic group?	1= Chewa<br>2= Tumbuka<br>3= Lomwe<br>4= Tonga<br>5= Yao<br>6= Sena<br>7= Nkonde<br>8= Ngoni<br>99= Others (specify):	ETHICHH	
5	How many persons live in your household?		HSHMEMNO	
6	How many children under 2 years do you have?		NOUNDER	
7	Literacy of the mother/caretaker	1= unable to read or write<br>2= able to read<br>3= able to read and write	LITMOTH	
8	What is the highest level of school you completed: primary, secondary, or higher?	1= Primary<br>2= Secondary<br>3= Higher<br>6= None	EDUCMOTH	
9	What is your main occupation (mention one or two with priority):	1= Farming<br>2= Business<br>3= Trades/vocational skills<br>4= Casual labour<br>5= Wage employment<br>6= None	JOBMOTH1<br>JOBMOTH2	

10	Literacy of the father	1= unable to read or write<br>2= able to read<br>3= able to read and write	LITFATH	
11	What is the highest level of school your husband/partner attended: primary, secondary, or higher?	1= Primary<br>2= Secondary<br>3= Higher<br>6= None	EDUCFATH	
12	Main occupation of the father (mention one or two with priority):	1= Farming<br>2= Business<br>3= Trades/vocational skills<br>4= Casual labour<br>5= Wage employment<br>6= None	JOBFATH1<br>JOBFATH2	

13	What was the main source of income of your HH during the last 4 weeks?	1= no income source<br>2= sale of home grown crops<br>3= sale of home reared livestock products<br>4= sale of fattening animals<br>5= sale of homemade crafts<br>6= sale of firewood/charcoal<br>7= waged labour<br>8= petty trade small business<br>9= loan<br>10= remittance<br>11= safety net labour<br>12= employment/salary<br>99= other (specify)	INCOME	

Living Conditions

14	How many rooms in your HH are used for sleeping?	Number of rooms	NOROOM	
15	What is the main material of the dwelling floor?	**1= Natural floor:**<br>Earth/sand, Dung<br>**2= Rudimentary floor:**<br>Wood planks, Palm/bamboo<br>**3= Finished floor:**<br>Parquet or polished wood, Vinyl or asphalt strips, Ceramic tiles, Cement, Carpet<br>**99= Other** (specify):	MATFLO	
16	What is the main material of the roof?	**1= Natural roofing:**<br>Thatch/palm leaf, Sod<br>**2= Rudimentary Roofing:**<br>Rustic mat, Palm/bamboo, Wood planks<br>**3= Finished roofing:**<br>Metal, Wood, Calamine/cement fiber, Ceramic tiles, Cement, Roofing shingles<br>**99= Other** (specify):	MATROOF	
17	What is the main material of the walls?	**1= Natural walls:**<br>No walls, Cane/palm/trunks, Dirt<br>**3= Rudimentary walls:**<br>Bamboo with mud, Stone with mud, Uncovered adobe, Plywood, Carton, Reused wood<br>**3= Finished walls:**<br>Cement, Stone with lime/cement, Bricks, Cement blocks, Covered adobe, Wood planks/shingles<br>**99= Other** (specify):	MATWALL	
18	What type of fuel does your HH mainly use for cooking?	1= Agricultural crop residue<br>2= Animal dung<br>3= Straw/shrubs/grass<br>4= Wood<br>5= Charcoal<br>6= Coal / Lignite<br>7= Kerosene<br>8= Biogas<br>9= Natural gas<br>10= Liquid Propane Gas (LPG)<br>11= Electricity<br>99= Other (specify):	COKENER	
19	If firewood:<br>Who usually goes to collect firewood in your HH?	1= yourself<br>2= family member ➔Q 21	FIRECOLL	
20	How long does it take to collect firewood and come back?	Number of minutes ___________<br>88= don't know	FIRETIME	

21	What is the main source of drinking water for members of your HH?	**1= Piped water:**<br>Piped into dwelling, yard or plot, Public tap/standpipe, Tubewell / borehole with hand-pump, with powered pump<br>**2= unimproved Dug well / spring:**<br>Unprotected well, unprotected spring<br>**3= improved Dug well / spring:**<br>Protected well, protected spring<br>4= Rainwater collection<br>5= Tanker-truck, Cart with small tank/drum<br>**6= Surface water:**<br>river, stream, dam, lake, pond, canal, irrigation channel<br>7= Bottled water<br>99 = other (specify):	WATDRINK	
22	What is the main source of water used by your HH for other purposes such as cooking & hand washing?	**1= Piped water:**<br>Piped into dwelling, yard or plot, Public tap/standpipe, Tubewell / borehole with hand-pump, with powered pump<br>**2= unimproved Dug well / spring:**<br>Unprotected well, unprotected spring<br>**3= improved Dug well / spring:**<br>Protected well, protected spring<br>4= Rainwater collection<br>5= Tanker-truck, Cart with small tank/drum<br>**6= Surface water:**<br>river, stream, dam, lake, pond, canal, irrigation channel<br>99 = other (specify):	WATCOOK	
23	If you do not have a water source at your premises:<br>Who usually goes to fetch the water for your HH?	1= yourself<br>2= family member ➔ Q 25	WATFETCH	
24	How long does it take to collect water and come back?	Number of minutes ___________<br>88= don't know	WATTIME	
25	How do you store the water in the HH?	1= Jerry can/Narrow neck container with lid<br>2= Jerry can/Narrow neck container without lid<br>3= Open container with lid<br>4= Open container without lid<br>99= Others (specify)	WATSTO	
26	Do you treat your water in any way to make it safer to drink?	1= yes<br>2= no ➔**Q 28**<br>88= don't know	WATSAFE	
27	What do you usually do to the water to make it safer to drink?<br>Anything else? (Record all items mentioned)	1= Boil<br>2= Add bleach/chlorine<br>3= Strain it through a cloth<br>4= Use water filter (ceramic, sand, composite, etc.)<br>5= Solar disinfection<br>6= Let it stand and settle<br>99= Other (specify)<br>88= don't know	WATTREAT	
28	What kind of toilet facility do members of your HH usually use?<br>If "flush" or "pour flush": probe where does it flush to?	**1= unimproved Flush / pour flush**<br>Flush to somewhere else<br>**2= improved Flush / pour flush:**<br>Flush to piped sewer system, Flush to septic tank, Flush to pit (latrine), Flush to unknown place, not known where<br>**3= unimproved Pit latrine:**<br>Pit latrine without slab/open pit,<br>**4= improved Pit latrine:**<br>Ventilated Improved Pit latrine (VIP), Pit latrine with slab, Pit latrine with slab & cover, Pit latrine with slab, cover & foot rest<br>**5= Composting toilet**<br>**6= Bucket**<br>**7= Hanging toilet/hanging latrine**<br>**8= No facilities or bush or field**<br>**99= Other** (specify)	LATRINE	

29	Does your HH have soap (or washing powder/ liquid) at present?	1= yes<br>2= no	LATSOAP	
30	How is garbage / household waste disposed of usually?	1= disposed openly in the street/garden/field<br>2= burried<br>3= burned<br>4= compost<br>99= other specify	GARBAGE1<br><br>GARBAGE2	
31	Which are the most common illnesses in your family?<br>*(don't read the answers!! mark according to the priority/order of their answers!)*	1= malaria<br>2= diarrhoea<br>3= respiratory tract diseases (ARI)<br>4= internal parasite<br>5= fever<br>6= restless/mental stress<br>7= injuries/wounds<br>99= others ____________________	HPROBL1<br><br>HPROBL2<br><br>HPROBL3	
32	How long does it take to walk to the nearest health facility for treatment? (one way)	1= less than 30 minutes<br>2= more than 30 minutes less than 1 hour<br>3= between 1 and 2 hours<br>4= more than 2 hours	TIMEHEAL	
33	Have you heard of the FAO/FICA project?	1= Yes<br>2= No	FAOFICA	
34	Has your household been a beneficiary?	1= Yes<br>2= No<br>88= Don't know	FICABENE	
35	In which FICA activities has your household been involved?	1= Irrigation<br>2= Seed distribution<br>3= Livestock distribution<br>4= Farmer Field Schools<br>99= Others (specify):	FICAACT	

Household Dietary Diversity

36	Please describe the foods (meals and snacks) that you ate yesterday during the day and night, whether at home or outside the home. Start with the first food eaten in the morning			
	**Food group**	**Examples**	1= yes<br>2= no	
	Cereals	bread, noodles, biscuits, cookies or any other foods made from millet, sorghum, maize, rice, wheat + *insert local foods e.g. nsima, porridge or pastes or other locally available grains*	HHDD1	☐
	Vitamin A rich vegetables and tubers	pumpkin, carrots, squash, or sweet potatoes that are orange inside + *other locally available vitamin-A rich vegetables(e.g. sweet pepper)*	HHDD2	☐
	White tubers and roots	white potatoes, white yams, cassava, or foods made from	HHDD3	☐
	Dark green leafy vegetables	dark green/leafy vegetables, including wild ones + *locally available vitamin-A rich leaves such as cassava leaves etc.*	HHDD4	☐
	Other vegetables	other vegetables (e.g. tomato, onion, eggplant) , including wild vegetables	HHDD5	☐
	Vitamin A rich fruits	ripe mangoes, cantaloupe, dried apricots, dried peaches + *other locally available vitamin A-rich fruits*	HHDD6	☐
	Other fruits	other fruits, including wild fruits	HHDD7	☐
	Organ meat (iron rich)	liver, kidney, heart or other organ meats or blood-based foods	HHDD8	☐
	Flesh meats	beef, pork, lamb, goat, rabbit, wild game, chicken, duck, or other birds	HHDD9	☐
	Eggs		HHDD10	☐
	Fish	fresh or dried fish or shellfish	HHDD11	☐
	Legumes, nuts and seeds	beans, peas, lentils, nuts, seeds or foods made from these	HHDD12	☐
	Insects	insect larvae, lake fly, ants	HHDD13	☐
	Milk and milk products	milk, cheese, yogurt or other milk products	HHDD14	☐
	Oils and fats	oil, fats or butter added to food or used for cooking	HHDD15	☐
	Sweets	sugar, honey, sweetened soda or sugary foods such as chocolates, sweets or candies	HHDD15	☐
	Spices, condiments, beverages	Spices (black pepper, salt), condiments (soy sauce, hot sauce), coffee, tea, alcoholic beverages OR *local examples*	HHDD16	☐

Household food security

38	***READ TO RESPONDENT***: "For each of the following questions, consider whether this has happened in the past 4 weeks. If the answer is yes to a question, please indicate how often this happened."			
	In the past [4 weeks], did you worry that your household would not have enough food?	0= No, did not happen in the past [4 weeks]<br><br>Yes, did happen in the past [4 weeks]<br>1= Rarely, 1-2 times<br>2= Sometimes, 3-10 times<br>3= Often, more than 10 times	FOODSEC1	☐
	In the past [4 weeks], did it happen that you or any household member were not able to eat the kinds of foods you would have preferred to eat because of lack of resources?	0= No, did not happen in the past [4 weeks]<br><br>Yes, did happen in the past [4 weeks]<br>1= Rarely, 1-2 times<br>2= Sometimes, 3-10 times<br>3= Often, more than 10 times	FOODSEC2	☐
	In the past [4 weeks], did it happen that you or any household member had to eat a limited variety of foods because of lack of resources?	0= No, did not happen in the past [4 weeks]<br><br>Yes, did happen in the past [4 weeks]<br>1= Rarely, 1-2 times<br>2= Sometimes, 3-10 times<br>3= Often, more than 10 times	FOODSEC3	☐
	In the past [4 weeks] did it happen that you or any household member had to eat some foods that you really did not want to eat because of lack of resources?	0= No, did not happen in the past [4 weeks]<br><br>Yes, did happen in the past [4 weeks]<br>1= Rarely, 1-2 times<br>2= Sometimes, 3-10 times<br>3= Often, more than 10 times	FOODSEC4	☐
	In the past [4 weeks] did it happen that you or any household member had to eat a smaller meal than you felt you needed because there was not enough food?	0= No, did not happen in the past [4 weeks]<br><br>Yes, did happen in the past [4 weeks]<br>1= Rarely, 1-2 times<br>2= Sometimes, 3-10 times<br>3= Often, more than 10 times	FOODSEC5	☐
	In the past [4 weeks] did it happen that you or any household member had to eat fewer meals in a day because there was not enough food?	0= No, did not happen in the past [4 weeks]<br><br>Yes, did happen in the past [4 weeks]<br>1= Rarely, 1-2 times<br>2= Sometimes, 3-10 times<br>3= Often, more than 10 times	FOODSEC6	☐
	In the past [4 weeks] did it happen that there was no food to eat of any kind in your house, because of lack of resources to get food?	0= No, did not happen in the past [4 weeks]<br><br>Yes, did happen in the past [4 weeks]<br>1= Rarely, 1-2 times<br>2= Sometimes, 3-10 times<br>3= Often, more than 10 times	FOODSEC7	☐
	In the past [4 weeks] did it happen that you or any household member<br>went to sleep at night hungry because there was not enough food?	0= No, did not happen in the past [4 weeks]<br><br>Yes, did happen in the past [4 weeks]<br>1= Rarely, 1-2 times<br>2= Sometimes, 3-10 times<br>3= Often, more than 10 times	FOODSEC8	☐
	In the past [4 weeks] did it happen that you or any household member<br>went a whole day and night without eating anything at all because there was not enough food?	0= No, did not happen in the past [4 weeks]<br><br>Yes, did happen in the past [4 weeks]<br>1= Rarely, 1-2 times<br>2= Sometimes, 3-10 times<br>3= Often, more than 10 times	FOODSEC9	☐
	What is currenty the main source of food for your household?	1= Own Food Production<br>2= Purchased Food<br>3= Borrowed Food<br>4= Food Gift<br>5= Food Aid<br>6= Ganyu<br>7= Food for work<br>99= Other (specify):	FOODSOU	☐☐

Possessions and income

39	*Does your household have access to or own…..:*		1= yes<br>2= no	
	Electricity?		POSSEL	☐
	Radio?		POSSRA	☐
	Television?		POSSTV	☐
	Mobile Telephone?		POSSMP	☐
	Refrigerator?		POSSFR	☐
	Bicycle?		POSSBIC	☐
	Motorcycle?		POSSMO	☐
	Car or truck?		POSSCAR	☐
41	Does any member of this household own any land that can be used for agriculture?	1= Yes<br>2= No	HHLAND	☐
42	How many hectares of agricultural land do members of this household own?<br>If more than 97, record '97'.<br>If unknown, record '88'.	Acres:<br><br>(Or) Hectares:	LANDACR<br><br>LANDHEC	☐<br><br>☐
43	Do you have a home garden?	1=yes<br>2=no	HOMEGAR	☐
44	Do you grow vegetables?	1=yes<br>2=no	GARVEG	☐
45	Do you grow fruits?	1=yes<br>2=no	GARFRUIT	☐
46	Main use of vegetable products	1= Consumption<br>2= Sales<br>3= Gift<br>99= Other specify:	USEVEG	☐☐
47	Does this HH own any livestock, herds, or farm animals?	1=yes<br>2=no	ANIMALS	☐
48	*How many of the following animals does your HH have?*<br>If none, record '00', If more than 97, record '97', If unknown, record '88'			
	Cattle?		CATTLE	☐☐
	Horses, donkeys, or mules?		HORSE	☐☐
	Goats?		GOAT	☐☐
	Sheep?		SHEEP	☐☐
	Pigs?		PIG	☐☐
	Chickens?		CHICK	☐☐
49	For what reason do you keep animals?	1= mainly own consumption<br>2= mainly for sale<br>3= both (in approx. equal amounts)	REAANIM	☐☐

QUESTIONNAIRE for Children under 2 years

Identity Number of the household		District EPA	Village no	Family	IDNO Child
1	Do you have a record of your child's date of birth?<br>*Please ask other household members to confirm the date*	*If yes, please record*: month, day, year<br>*and proceed*➔ Q 2<br>If not, *please proceed* ➔ Q 1a	BIRTHDAT		
1a	*Present the local calendar and ask:*<br>Do you remember a local event when your child was born?	*Record which sandwich could be isolated and record: month, year.*<br>In case of 1 month please proceed ➔ Q 2<br>!! In case of 2 month please thank the mother/ caretaker for her time and close the interview	BIRTHSEAC		
Please note: If child age is over 23 months thank the mother for her time and end interview.					
2	Is your child a boy or a girl?	1= male<br>2= female	CHSEX		
3	Does your child have a CHDR (Health Card)?	1= yes, available ➔ Q 3a<br>2= yes, but not available ➔ Q 4<br>3= no ➔ Q 4	HEALCARD		
3a	*Copy the birth weight of the child (in g)*		BIRTHWEI		
4	Is there a vaccination card of the child available?<br>*If vaccination card available please record directly from card.*	1= yes, seen<br>2= yes, not seen<br>3= no	VACCARD		
5	Has (Name) ever been given a BCG vaccination against tuberculosis – that is, an injection in the arm or shoulder that caused a scar?	1= yes<br>2= no<br>88= don't know	BCG		
6	Has (Name) ever been given any polio vaccination drops in the mouth?	0= never<br>1= once<br>2= twice<br>3= more than twice<br>88= don't know	OPV		
7	Has (Name) ever been given "DPT 1-3" – that is, an injection in the thigh or buttocks?	0= never<br>1= once<br>2= twice<br>3= three times or more<br>88= don't know	DPT		
8	Has (Name) ever been given "Measles vaccination injections" or MMR – that is, a shot in the arm at the age of 9 months or older?	1= yes<br>2= no<br>88= Don't know	MEASLES		
9	Has (Name) taken any drug for intestinal worms in the last 6 months?	1= yes<br>2= no<br>88= don't know	DEWORM		
10	Within the last six months was (name) given a vitamin A dose like any of these?<br>*Show common types of ampules/ capsules/ syrups*	1= yes<br>2= no<br>88= don't know	VIT A		
11	Has (Name) had fever in the past two weeks?	1= yes<br>2= no<br>88= don't know	CHFEV		
12	Has (Name) had diarrhoea in the past two weeks, that is, since (day of the week) of the week before last?<br>Diarrhoea is determined as perceived by mother or caretaker, or as three or more loose or watery stools per day, or blood in stool.	1= yes<br>2= no<br>88= don't know	CHDIAR		
13	Has (Name) had an illness with a cough at any time in the past two weeks, that is, since (day of the week) of the week before last?	1= yes<br>2= no<br>88= don't know	CHARI		

14	When (Name) had an illness with a cough, did he/ she breathe faster than usual with short, quick breaths or have difficulty breathing?	1= yes<br>2= no<br>88= don't know	CHARIBR	
15	Were the symptoms due to a problem in the chest or a blocked nose?	1= problem in chest<br>2= blocked nose<br>3= both<br>99= other (specify)<br>88= don't know	CHARISYM	
16	Has (Name) had fever with chills (Malaria) in the past two weeks?	1= yes<br>2= no<br>88 = don't know	CHMAL	

Breast feeding and complementary feeding practices

17	Did you ever breastfeed (Name)?	1= yes<br>2= no	BFEVER	
18	Did (name) received the first milk (colostrum)?	1= yes<br>2= no<br>88 = don't know	RECCOL	
19	What was the first food that (Name) was given apart from breast milk?<br>*Read each item aloud and record response before proceeding to the next item.*		1= yes<br>2= no<br>88= don't know	
	Plain water?		REWATER	
	Sweetened/flavoured water?		RESWEET	
	Tea or infusion?		RETEA	
	Fruit juice?		REJUICE	
	Infant formula?		REIF	
	Tinned, powdered or fresh milk?		REMILK	
	Other liquids (specify)?		RELIQU	
	Porridge?		REPOR	
	Mashed food?		REFOOD	
20	Is he/she still being breastfed?	1= yes ➔ Q 23<br>2= no	BFSTILL	
21	If the child is not breastfed anymore, at what age (in month) did you stop breastfeeding?	*Record age in months*<br>(please verify by asking other household members and by using the local calendar)	BREASTOP	
22	Why did you stop breastfeeding?	1= not enough milk<br>2= feel too weak<br>3= wanted to stop (child old enough)<br>4= no time to breastfeed<br>5= pregnancy<br>99= others	WHYSTOP	
23	Was (name) breastfed yesterday during day or at night?	1= yes<br>2= no<br>88= don't know	BFYESTER	
24	Did (name) consume breast milk in any other way yesterday during the day or at night?<br>e.g. by spoon, cup or bottle; by his/her mother or another woman	1= yes<br>2= no<br>88= don't know	BFNURSE	

25	Since this time yesterday, did he/she receive any of the following:<br>*Read each item aloud and record response before proceeding to the next item.*		1= yes<br>2= no<br>88= don't know	
	Plain water?		CHREWAT	
	Sweetened, flavored water or fruit juice or tea or infusion?		CHREJUIC	
	Vitamin, mineral supplements or medicine?		CHREVIT	
	Oral rehydration solution (ORS)?		CHREORS	
	Infant formula?		CHREIF	
	Tinned, powdered or fresh milk?		CHREMILK	
	Any other liquids?		CHRELIQU	
26	At what age did you start giving (Name) other food apart from breast milk?	*Record age in months*<br>(please verify by asking other household members and by using the local calendar)	CFAGE	
27	Did (Name) receive solid, semi-solid or soft food yesterday?	1= yes<br>2= no ➔ Q 30<br>88= don't know ➔ Q 30	CHRFOOD	

28	What kind of food did (Name) receive yesterday during day or night?<br>*Read each item aloud and record response before proceeding to the next item.*	1= yes<br>2= no<br>88= don't know	
	Porridge, bread, rice, noodles, or other foods made from grains	A	
	Pumpkin, carrots, squash, or sweet potatoes that are yellow or orange inside	B	
	White potatoes, white yams, manioc, cassava, or any other foods made from roots	C	
	Any dark green leafy vegetables	D	
	Ripe mangoes, ripe papayas, or… local vit A rich foods	E	
	Any other fruits or vegetables	F	
	liver, kidney, heart, or other organ meats	G	
	Any meat, such as beef, pork, lamb, goat, chicken, or…	H	
	Eggs	I	
	Fresh or dried fish, shellfish, or seafood	J	
	Any foods made from beans, peas, lentils, nuts, or seeds	K	
	Cheese, yogurt or other milk products	L	
	Any fat, oil or butter or foods made with any of these	M	
	Any sugary foods such as chocolates, sweets, candies, pastries, cakes, or biscuits	N	
	Condiments for flavor, such as chilies, spices, herbs, or fish powder	O	
	Insects	P	

29	Yesterday, during the day or night, did (NAME) consume any food to which you added:<br>*Read each item aloud and record response before proceeding to the next item.*		1= yes<br>2= no<br>88= don't know	
	Powder or sprinkles?		CFSPRK	
	Fat based nutrient supplement?		CFLIPID	
	Iron fortified infant/toddler formula?		CFIRON	
30	*If child receives food:* How many times did (Name) receive food yesterday?	Number of times<br>88= don't know	FEEDFQ	
31	*If child is breastfed:* How many times was (Name) breastfed yesterday?	Number of times<br>88= don't know	BFFREQ	
32	Did you prepare special meals for (Name) yesterday?	1= yes<br>2= no	SPMEAL	
33	What prevented you to prepare special meals for (Name) yesterday?	1= don't know how to do<br>2= lack of time<br>3= miss food<br>99= other (specify)	SPMPREV	

QUESTIONNAIRE for MOTHER

Identity Number of the household	District EPA	Village no	Family

1	Age of the mother in years (approximately)		AGEMOTH	
2	Are you pregnant at the moment?	1= yes<br>2= no	PREGNANT	
3	Do you have any food restrictions?	1= yes<br>2= no → 4	RESPREG	
3a	Please specify your food restrictions: *(ask especially for food restrictions during pregnancy and lactating)*			
4	Do you follow any fasting rules	1= yes<br>2= no → 5	FASTPREG	
4a	Please specify your fasting rules: *(ask especially for fasting during pregnancy and lactating)*			
5	During your last pregnancy and/or lactating period did you use CSB?	1= yes<br>2= no	USECSB	
6	During your last pregnancy and/or lactating period did you receive any supplements?	1= yes (specify):<br>2= no	SUPPLEM	
7	Do you use iodised salt for cooking?	1= yes<br>2= no	IODSALT	
8	Do you know what causes night blindness?	1= yes<br>2= no	DEFVITA	
8a	Please name 3 foods that are rich in vitamin A	1= able to name 3 foods<br>2= unable to name 3 foods	FOODVITA	
8b	What is the color of vitamin A rich foods?	1= correct answer: orange<br>2= wrong answer	COLVITA	
9	Do you know what causes anemia?	1= yes<br>2= no	DEFIRON	
9a	Please name 3 foods that are rich in iron	1= able to name 3 foods<br>2= unable to name 3 foods	FOODIRON	
10	If your child had fever in the past 2 weeks, what did you do?	1= traditional medicine<br>2= go to clinic<br>3= use local herbs at home<br>4= buy drugs in market<br>5= seek advice from health worker<br>6= nothing<br>99= other (specify)	FEVTREAT	
11	If your child had diarrhea in the past 2 weeks, what did you do?	1= traditional medicine<br>2= go to clinic<br>3= use local herbs at home<br>4= buy drugs in market<br>5= seek advice from health worker<br>6= ORS<br>7= give more fluids<br>8= nothing<br>99= other (specify)_________________	DIATREAT	
12	How often do you offer fluids when (Name) is ill?	1= more than usual<br>2= less than usual<br>3= same as usual	ILLFLUID	
13	How often do you offer the breast when (Name) is ill?	1= more than usual<br>2= less than usual<br>3= same as usual	ILLBREAST	

14	How often do you offer food when (Name) is ill?	1= more than usual<br>2= less than usual<br>3= same as usual	ILLFOOD	
15	From whom have you received infant feeding support or assistance?<br>*Do not read out the list, probe for further responses. More than one answer possible.*	1= yes<br>2= no		
	Health worker		IFSUPP1	
	NRU/Health poste		IFSUPP2	
	Mother		IFSUPP3	
	Mother in law		IFSUPP4	
	Grandmother		IFSUPP5	
	Chief		IFSUPP6	
	Friend/neighbor		IFSUPP7	
	99= Other (specify)		IFSUPP8	
16	Who decides how you would feed your baby?<br>*Do not read out the list, probe for further responses. More than one answer possible.*	1= yes<br>2= no		
	Myself		IFDECI1	
	Husband/partner		IFDEC2	
	Grandmother		IFDEC3	
	Mother		IFDEC4	
	Mother in law		IFDEC5	
	99= Other (specify)		IFDEC6	

17	How can you help preventing getting malaria?<br>*Do not read out the list, probe for further responses. More than one answer possible.*	1= yes<br>2= no		
	Use an ITN		PREVMA1	
	Cover up at dusk		PREVMA2	
	IRS		PREVMA3	
	Removal of mosquito breeding sites		PREVMA4	
	99= Other (specify)		PREVMA6	
18	What causes diarrhea?<br>*Do not read out the list, probe for further responses. More than one answer possible.*	1= yes<br>2= no		
	Contaminated food		CUASDIA1	
	Contaminated water		CAUSDIA2	
	Contaminated hands		CAUSDIA3	
	Flies		CAUSDIA4	
	Eating greens		CAUSDIA5	
	99= Other (specify)		CAUSDIA6	

19	How can you help to prevent diarrhea?<br>*Do not read out the list, probe for further responses. More than one answer possible.*	1= yes<br>2= no		
	Washing hands		PREVDIA1	
	Use latrine or bury faces		PREVDIA2	
	Boil drinking water		PREVDIA3	
	Exclusive breast feeding		PREVDIA4	
	99= Other (specify)_________		PREVDIA5	
20	Have you used soap yesterday or today?	1= yes<br>2= no<br>3= do not have soap	SOAPYEST	
21	When you used soap today or yesterday, what did you use it for?<br>If for washing my hands are mentioned, probe what was the occasion, but do not read the answers!<br>*(Do not read the answers, ask to be specific, encourage "what else" until nothing further is mentioned and check all that apply)*			
	Washing clothes	A	WCHLOTH	
	Washing my body	B	WBODY	
	Washing my children	C	WCHILD	
	Washing child's bottoms	D	WCHILDB	
	Washing my children's hands	E	WCHILDH	
	Washing hands after defecating	F	WCHILDD	
	Washing hands after cleaning child	G	WAFTERC	
	Washing hands before feeding child	H	WBEFFED	
	Washing hands before preparing food	I	WBEFFOOD	
	Washing hands before eating	J	WBEFEAT	
	99= Other(Specify)_________________		WOTHER	
22	On average how much time do you spend for household chores per day?	Time in hours:	TIMEHHC	
23	Do you work outside your homestead?	1= yes<br>2= no	WORKOUT	
24	Who is taking care of your child when you have to go out?	1= I am taking it with me<br>2= father<br>3= elder children<br>4= grandmother<br>99= other (specify)	CARECH1	
25	Who is taking care of your children when you are sick?	1= father<br>2= elder children<br>3= grandmother<br>4= friends/neighbors<br>99= other (specify)	CARECH2	
26	Within the last month have you been seriously ill?<br>*Please explain "seriously" as too ill to work or take care of her children*	1= yes → 26<br>2= no	TIMEILL	
27	Please explain your illness and the major symptoms, how often do you suffer from this diseases?			

Informed Consent Form for Mothers/Caretakers

My name is ___________ and I work as an enumerator in the IMCF research project. We are conducting a research on "Improving the dietary intakes and nutritional status of infants and young children through improved food security and complementary feeding counseling" (IMCF). This study will provide knowledge on infant and young child feeding practices that may be used to improve nutrition programs for infants and young children.

What you will be asked if you participate in the study? If you decide to participate in this study, an interviewer will ask a series of questions about your child, (name of the child:____________) that will take about one hour. For example, the interviewer will ask you about some household characteristics such as your name and your husband's name and practices about infant and young child feeding--the age at which (name of the child:___________) was first offered foods other than breast milk, how many times a day your child eats, how much of each food your child eats, which foods your child prefers, how you prepare the foods for your child, and what is the normal consistency of the foods you offer to your child.

The anthropometric measurements of you and your child will be taken at a central measuring station. The research staff will ensure your privacy. You will be asked to remove your shoes and heavy clothing to measure your weight. Your child's weight will be taken while you hold him or her on your arms with very light clothing to ensure accuracy. Your child's length will be taken while lying down and the researchers will do their best to create a peaceful environment. Additionally, a blood sample from your finger pad and your child's finger pad will be taken by a professional health personal to assess hemoglobin levels and vitamin A status.

Participation: You are being invited to take part in this research because you have been selected randomly among all mothers with young children in your community. Your participation in this research is entirely voluntary. It is your choice whether to participate or not. You can withdraw from the study at any time. If you withdraw, your data and information will be destroyed.

Risks and Discomforts: We are asking you to share some personal and confidential information with us, and you may feel uncomfortable talking about some of the topics. You do not have to answer any question if you do not wish to do so. You do not have to give us any reason for not responding to any question or for refusing to take part in the interview.

Regarding the collection of blood samples, the finger prick method is a quick and easy method, which may, however, make your child or yourself feel uncomfortable. To avoid infections the health personnel will disinfect the finger pad and use disposable finger pricks. Local compression will be used to prevent secondary hemorrhage. There are no further risks.

Benefits: There will be no direct benefit to you, but your participation will help us find out more information on how to prevent malnutrition of children and improve complementary feeding in your community.

Reimbursements: You will not be provided any incentive to take part in the research. However, we will give you a copy of your and your child's weight, height, calculated BMI and the results of your iron status.

Confidentiality: The information you will provide during the interview is strictly confidential, will only be available to the project investigators, and will not be provided to anyone else. To ensure confidentiality, each participate will be assigned a unique number that will be used instead of the name. Only investigators and supervisors in charge of the study will have access to records linking participants' names and numbers. If the results are published, your identity will remain confidential.

Who to Contact? If you have any questions about the study, you are welcome to ask the interviewer (name of interviewer) or the supervisor for the study (Name of supervisor) at any time. For further information, you can contact: Dr Beatrice Mtimuni (cell phone number: -0888-851-870). If you have any questions about rights of research subjects or research-related injury, please contact the National Health Science Research Committee (NHSRC)—(The Chairperson, phone number: 01 789 400/ 414).

Sincerely,
Dr. Beatrice Mtimuni (Study Coordinator)

I give consent for me and my son/daughter to participate in this research study which involves completing an interview, taking anthropometric measurements, and blood samples. I have had the opportunity to ask questions about it and any questions that I have asked have been answered to my satisfaction. I consent voluntarily for me and my child to participate in the study.

Print Name of Parent or Caretaker ______________________

Signature of Parent or Caretaker ______________________

Date __________________________
day/month/year

If illiterate

I have witnessed the accurate reading of the consent form to the parent/caretaker, and the individual has had the opportunity to ask questions. I confirm that the individual has given consent freely.

Print Name of witness ______________________ **and thumb print of participant**

Signature of witness ______________________

Date __________________________
day/month/year

I have accurately read out the information sheet to the parent/caretaker, and did the best of my ability to make sure that the person understood the following:

1. face-to-face interview
2. anthropometric measurements of mother/caretaker and child
3. taking blood samples of mother/caretaker and child

I confirm that the mother/caretaker was given an opportunity to ask questions about the study, and all the questions asked have been answered correctly and to the best of my ability. I confirm that the individual has not been coerced into giving consent, and the consent has been given freely and voluntarily.

Print Name of enumerator ______________________

Signature_____________________________________ Date______________________________

Identity Number of the household (District: Kasungu = K, Mzimba = M, EPA, No. of Village)	☐☐ ☐☐ District EPA	☐☐☐☐ Village no	☐☐☐☐ Family

I give consent for me and my son/daughter to participate in this research study which involves completing an interview, taking anthropometric measurements, and blood samples. I have had the opportunity to ask questions about it and any questions that I have asked have been answered to my satisfaction. I consent voluntarily for me and my child to participate in the study.

Print Name of Parent or Caretaker ______________________

Signature of Parent or Caretaker ______________________

Date __________________________
day/month/year

If illiterate

I have witnessed the accurate reading of the consent form to the parent/caretaker, and the individual has had the opportunity to ask questions. I confirm that the individual has given consent freely.

Print Name of witness ______________________ **and thumb print of participant**

Signature of witness ______________________

Date __________________________
day/month/year

I have accurately read out the information sheet to the parent/caretaker, and did the best of my ability to make sure that the person understood the following:

1. face-to-face interview
2. anthropometric measurements of mother/caretaker and child
3. taking blood samples of mother/caretaker and child

I confirm that the mother/caretaker was given an opportunity to ask questions about the study, and all the questions asked have been answered correctly and to the best of my ability. I confirm that the individual has not been coerced into giving consent, and the consent has been given freely and voluntarily.

A copy of this Informed Consent Form has been provided to the mother/caretaker.

Print Name of enumerator ______________________

Signature____________________________________ Date______________________________
